# Propolis in Obesity and Related Metabolic Disorders: Mechanistic and Clinical Insights—A Scoping Review

**DOI:** 10.3390/nu18050826

**Published:** 2026-03-03

**Authors:** Kadriye Elif İmre, Aslı Akyol

**Affiliations:** 1 Department of Nutrition and Dietetics, Kastamonu University, 37150 Kastamonu, Turkey; keimre@kastamonu.edu.tr; 2 Department of Nutrition and Dietetics, Hacettepe University, 06100 Ankara, Turkey

**Keywords:** propolis, obesity, metabolic disorders, insulin resistance, dyslipidemia, non-alcoholic fatty liver disease, gut microbiota, inflammation

## Abstract

Objectives: Obesity and related metabolic disorders, including insulin resistance, dyslipidemia, and non-alcoholic fatty liver disease, represent major global health challenges. Growing interest in complementary strategies has brought propolis, a resinous bee-derived product rich in phenolic and flavonoid compounds, into focus. This scoping review aimed to map and synthesize available in vitro, in vivo, and clinical evidence regarding the metabolic effects of whole propolis preparations and propolis-derived bioactive compounds in obesity-related contexts. Methods: The review was conducted in accordance with the PRISMA-ScR framework and included experimental and human studies evaluating adipogenesis, lipid and glucose metabolism, oxidative stress, inflammatory signaling, non-alcoholic fatty liver disease-related outcomes, and gut microbiota modulation. Results: Across preclinical models, propolis preparations have been associated with modulation of antioxidant defenses, attenuation of inflammatory signaling, regulation of adipogenic transcriptional programs, and alterations in gut microbiota composition and barrier integrity. Clinical evidence suggests modest improvements in selected metabolic and inflammatory biomarkers; however, effects on body weight and adiposity remain inconsistent. Interpretation is limited by heterogeneity in propolis type, extraction method, chemical standardization, dosing strategies, and study design. Conclusions: Overall, current evidence indicates that propolis may influence obesity-related metabolic pathways, primarily at the level of biomarker modulation. Nevertheless, mechanistic causality and long-term clinical efficacy require confirmation through well-designed, adequately powered, and chemically standardized trials.

## 1. Introduction

The World Health Organization describes obesity as an excessive build-up of body fat that can endanger health. More than one billion people are currently affected worldwide [1]. Projections suggest that by the year 2050, more than half of the global adult population aged 25 years and older, approximately 3.8 billion people, along with nearly one-third of children and adolescents, equating to around 746 million individuals, will be classified as overweight or obese [2]. Obesity develops as a result of the combined influence of genetic factors, social and economic conditions, and cultural lifestyle patterns. It is commonly seen alongside other chronic health problems, including type 2 diabetes, hypertension, and cardiovascular disease, and plays a significant role in both the onset and progression of these comorbid conditions [3].

Beyond its epidemiological impact, obesity is also characterized by complex molecular and cellular changes that drive disease progression. In obesity, excess fat does not accumulate in isolation; dysfunctional adipose tissue also triggers wide-ranging metabolic disturbances. Hypertrophic adipocytes and tissue hypoxia trigger the recruitment of pro-inflammatory M1 macrophages into adipose tissue and the release of cytokines such as tumor necrosis factor-α (TNF-α), interleukin-6 (IL-6), and monocyte chemoattractant protein-1 (MCP-1), thereby establishing a state of chronic low-grade inflammation [4]. These alterations promote systemic inflammation and oxidative stress, which in turn modulate the secretion of adipokines—including adiponectin, leptin, visfatin, resistin, and chemerin—that regulate insulin sensitivity, energy expenditure, lipid distribution, and metabolic homeostasis, thereby playing a central role in the development of insulin resistance and mitochondrial dysfunction [5,6]. Furthermore, excessive nutrient intake increases intracellular free fatty acid (FFA) and glucose load, which—together with inflammation and adipokine dysregulation—imposes metabolic stress on mitochondria, disrupting their normal function and accelerating the onset of obesity-related metabolic disorders [7].

The treatment of obesity relies on a range of evidence-based options. These include behavioral and dietary approaches, increased physical activity, drug therapy, and metabolic or bariatric surgery [8]. Lifestyle change is considered the foundation of most treatment plans. Even so, maintaining weight loss in the long term can be difficult for many individuals. Medications and surgical procedures can be effective when used appropriately, yet their application is often constrained by cost and potential side effects [3,8]. Because of these challenges, there is growing scientific interest in complementary strategies that make use of naturally occurring bioactive compounds. Among these, compounds with anti-inflammatory, antioxidant, and metabolic-modulating properties—such as those derived from plants and bee products—have attracted considerable attention for their potential to prevent and manage obesity and its related complications [9,10].

One of the natural products that has gained increasing scientific interest in this context is propolis. Honeybees collect plant resins and process them inside the hive to produce propolis, a complex substance rich in bioactive compounds, especially phenolics, which largely account for its pharmacological potential [11]. In particular, polyphenols derived from propolis have drawn growing attention due to their proposed therapeutic potential, including roles in metabolic regulation, immune modulation, and inflammation control [12]. Numerous studies have reported diverse biological activities of propolis, including its antioxidative, anti-inflammatory, antimicrobial, immunomodulatory, and neuroprotective effects—such as analgesic, anxiolytic, and antidepressant properties [13,14,15]. Traditionally, it has been applied in the treatment of inflammatory conditions, support of wound healing, and alleviation of various minor ailments [13,14].

Over the past decade, both in vitro and in vivo studies have expanded the understanding of the pharmaceutical potential of propolis in different health contexts [16,17,18]. Particular attention has been given to its polyphenolic components and their roles in weight regulation, including effects on adipocyte differentiation, lipid storage, and insulin signaling. Notably, anti-obesity effects have also been reported for other dietary polyphenols, including green tea catechins (e.g., EGCG), anthocyanins, curcumin, resveratrol, and quercetin, which have been linked to improvements in body weight and/or adiposity through modulation of lipid and energy metabolism pathways [12,19,20,21,22,23]. Despite valuable findings on the anti-obesity potential of polyphenols, important mechanistic questions remain unanswered. Resolving these issues will require comprehensive, well-designed experimental and clinical studies [12,14]. The aim of this review is to examine the metabolic effects of propolis, with a particular focus on its mechanisms in obesity-related disorders, and to highlight potential focal points for future research.

## 2. Methods

This scoping review aimed to map and summarize the available experimental and clinical evidence on propolis preparations and propolis-derived bioactive compounds in relation to obesity and obesity-related metabolic outcomes. The review was reported in accordance with the PRISMA-ScR checklist [24,25]. Reporting followed the PRISMA-ScR guideline [24].

We searched PubMed/MEDLINE, Scopus, and Web of Science Core Collection from inception to July 2025, without applying date restrictions. To reflect contemporary evidence, we prioritized studies published between January 2014 and July 2025, while earlier seminal articles were included when relevant. The search combined controlled vocabulary (when available) and free-text terms related to propolis and obesity/metabolic outcomes. The detailed PubMed/MEDLINE search strategy is provided in Supplementary Table S1. The final search was conducted on 12 July 2025.

We included (i) in vitro, (ii) in vivo animal, and (iii) human clinical studies assessing whole propolis preparations (e.g., ethanolic extract, aqueous extract, standardized preparations) and/or propolis-derived bioactive compounds (e.g., CAPE, chrysin, artepillin C) in relation to obesity or obesity-related metabolic outcomes (adiposity, body weight/BMI, insulin sensitivity, glycemic markers, lipid profile, inflammatory/oxidative biomarkers, non-alcoholic fatty liver disease (NAFLD)/metabolic dysfunction–associated steatotic liver disease (MASLD)-related outcomes, and gut microbiota/barrier endpoints). We also included relevant peer-reviewed reviews for background/contextualization. We excluded conference abstracts without full text, non-peer-reviewed items, studies not assessing propolis/propolis-derived interventions, and studies unrelated to obesity/metabolic outcomes.

**Figure 1:**
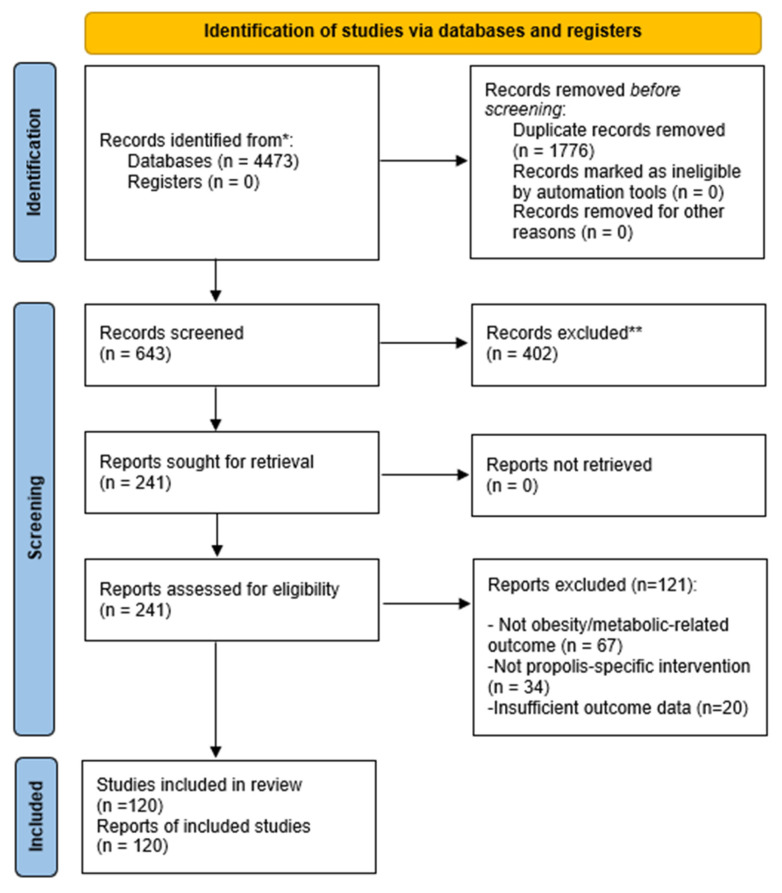
PRISMA-ScR 2020 flow diagram summarizing the identification, screening, eligibility assessment, and inclusion of studies. * Records identified from databases (PubMed/MEDLINE, Scopus, Web of Science Core Collection) and registers. ** Records excluded during title and abstract screening.

Records were exported to EndNote 22 (Clarivate Analytics, Philadelphia, PA, USA). After removal of duplicates, predefined database filters (language and document type) were applied prior to title and abstract screening. Titles and abstracts were then screened, followed by full-text assessment of potentially eligible articles. Title/abstract screening and full-text eligibility assessment were conducted by one reviewer and subsequently checked by a second reviewer. Reasons for full-text exclusion were recorded. The study selection process is summarized in the PRISMA-ScR flow diagram (Figure 1). Reference lists of included studies and relevant reviews were also screened to identify additional eligible articles.

We charted data on study design/model, population/sample, propolis type and extraction solvent (when reported), dose and duration, comparators, and outcomes (metabolic, inflammatory, oxidative stress, NAFLD/MASLD-related, and microbiota/barrier endpoints). Evidence was synthesized narratively and organized by evidence level (in vitro, in vivo, clinical) and outcome domain.

In line with scoping review methodology, we did not perform a formal risk-of-bias assessment [26]. Instead, we qualitatively considered reporting completeness (e.g., extract characterization/standardization, dose, duration, and outcome definitions) when interpreting findings.

## 3. Biochemical Composition and Key Bioactive Constituents of Propolis

Bees create propolis by collecting and modifying plant resins, producing a multifunctional material that supports hive defense and cohesion—hence its common name, ‘bee glue’ [27,28]. The term derives from Greek, meaning ‘in front of the city,’ a reference to its protective role for the hive [29]. Its therapeutic use dates back to ancient civilizations, with historical records documenting applications in Egyptian, Greek, and Roman cultures [13,29]. Hippocrates himself reportedly employed it in the treatment of wounds and ulcers [27,29]. From the 20th century onward, scientific interest in propolis intensified and gave rise to systematic investigations into its chemical composition and diverse biological activities [27].

Propolis contains over 180 identified chemical compounds, with its composition varying considerably according to season, geographic region, local flora, climate, bee species, and collection methods [30]. Harvested from both temperate and tropical regions, it has yielded more than 300 distinct constituents to date [31,32]. At room temperature, propolis exhibits a hard, brittle texture, but upon mild heating or handling, it softens into a sticky, malleable form, with a melting point of approximately 70 °C [33]. Its characteristic aromatic scent reflects a rich phytochemical profile—including polyphenols, terpenoids, amino acids, sugars, steroids, minerals, and vitamins—that underlies its broad spectrum of biological activities [12,16,17,18,30]. Among these, polyphenols—particularly flavonoids and phenolic acids—are regarded as the most pharmacologically relevant, with their abundance and composition determined largely by the botanical source [34].

Based on the botanical origin of the plant resins collected by bees, propolis can be classified into several major types, including poplar-type, Baccharis-type, Betula-type, Macaranga-type, and Dalbergia-type [12]. Each type displays a distinctive polyphenolic signature [30]. Poplar-type propolis, common in temperate regions such as Europe, North America, and parts of non-tropical Asia, is primarily derived from Populus species—including *Populus alba*, *Populus tremula*, and *Populus nigra* [35]. It is characterized by high levels of free phenolic acids (e.g., caffeic, *p*-coumaric, and ferulic acids) and their esters such as caffeic acid phenethyl ester (CAPE), in addition to flavonoids including chrysin, luteolin, apigenin, and kaempferol [36]. Baccharis-type propolis, prevalent in southern Brazil, Argentina, Uruguay, Paraguay, and Bolivia, originates primarily from the shrub *Baccharis dracunculifolia* [37]. This type is rich in prenylated phenolic compounds, with artepillin C and chlorogenic acid as defining bioactive constituents [38]. Dalbergia-type (red) propolis, derived from *Dalbergia ecastophyllum*, is notable for its diverse flavonoid composition, including flavanones, isoflavones, and dihydroflavonoids [38,39]. Propolis composition varies by botanical source and geographical origin, resulting in distinct chemical profiles across major propolis types (Table 1).

Propolis contains a broad spectrum of bioactive compounds. Key constituents include carboxylic acids, phenolic derivatives such as caffeic acid and its ester CAPE, cinnamic acid analogs, and terpenoids like pinocembrin and galangin. Additionally, saponins, phorbol esters, benzoic and coumaric acids, fatty acids, amino acids, apigenin, naringenin, naringin, gallic acid, steroids, vitamins, reducing sugars, and essential oils contribute to the complex pharmacological profile of propolis [44,45].

Flavonoids and phenolic acids in propolis act synergistically, contributing to its antioxidant, antimicrobial, and anti-inflammatory properties [46]. Their biological activities have been demonstrated in both in vitro and in vivo studies [31,47,48,49]. For instance, treatment with CAPE at concentrations ranging from 0.1 to 10 μM for 24 h significantly reduced reactive oxygen species (ROS) production and the expression of cell adhesion molecules in TNF-α-stimulated human endothelial cells [50]. In a rat model of lead-induced nephrotoxicity, oral administration of chrysin at daily doses of 25 and 50 mg/kg for seven days significantly attenuated oxidative damage and suppressed the expression of inflammatory markers [49]. Similarly, dietary supplementation with quercetin at 2 and 10 mg/kg/day for ten weeks improved insulin sensitivity and reduced hyperglycemia, dyslipidemia, and hepatic steatosis in obese Zucker rats [51]. Moreover, oral galangin at 50 mg/kg/day for six weeks in cafeteria diet-induced obese female rats significantly inhibited pancreatic lipase activity, thereby limiting fat absorption and body weight gain [52]. Collectively, these findings suggest that polyphenolic constituents of propolis may modulate obesity associated metabolic disturbances through multiple biological pathways [31].

Although propolis contains a wide spectrum of pharmacologically active constituents, its resinous and poorly soluble nature limits direct application in experimental and therapeutic contexts. To unlock its bioactive potential, extraction with suitable solvents—such as ethanol, methanol, water, hexane, acetone, dichloromethane, or chloroform—is required [53]. Among these, ethanol is most frequently employed, as it yields concentrated extracts comprising up to 70% propolis-derived material, thereby facilitating laboratory handling and comparative chemical analyses [54]. However, given the pronounced chemical heterogeneity of propolis across botanical and geographic origins, current consensus emphasizes standardization based on comprehensive phytochemical profiling rather than reliance on a single marker compound [28]. Such characterization is crucial for linking distinct chemical signatures to specific biological effects—particularly those relevant to complex metabolic disorders like obesity [54].

Propolis has been widely used in both traditional and modern practices. Although propolis has generally been well tolerated in short-term human studies, most human trials were short in duration and were not designed to evaluate long-term safety outcomes [55]. In addition, variability in botanical origin, extraction solvents, and formulation [56,57]. Importantly, the extraction solvent not only determines the chemical profile but may also influence tolerability and adverse-event patterns reported across studies. For example, ethanol, a solvent frequently employed in propolis extraction, has been associated with mild hepatic alterations following long-term exposure [58]. Rare allergic reactions such as contact dermatitis, most often involving the lips and oral mucosa, have also been documented in sensitive individuals [50,52]. Experimental animal studies consistently demonstrate low acute toxicity of propolis. Arvouet-Grand et al. (1993) reported an oral LD_50_ greater than 7340 mg/kg in mice, whereas Gritsenko et al. (1977) observed an LD_50_ of 2050 mg/kg and an LD_100_ of 2750 mg/kg [57,59]. In subchronic models, 90-day administration at 4600 mg/kg/day in mice elevated blood urea levels—attributed to the alcohol vehicle rather than propolis—whereas 1400 mg/kg/day produced no adverse histological or biochemical changes, indicating this dose as a potential no-observed-effect level (NOEL) [55]. Overall, available evidence suggests that propolis is generally well tolerated in experimental settings and short-term human studies; however, safety conclusions cannot be generalized across preparations, and long-term human safety data remain limited.

## 4. Therapeutic Mechanisms of Propolis Relevant to Obesity

**Figure 2:**
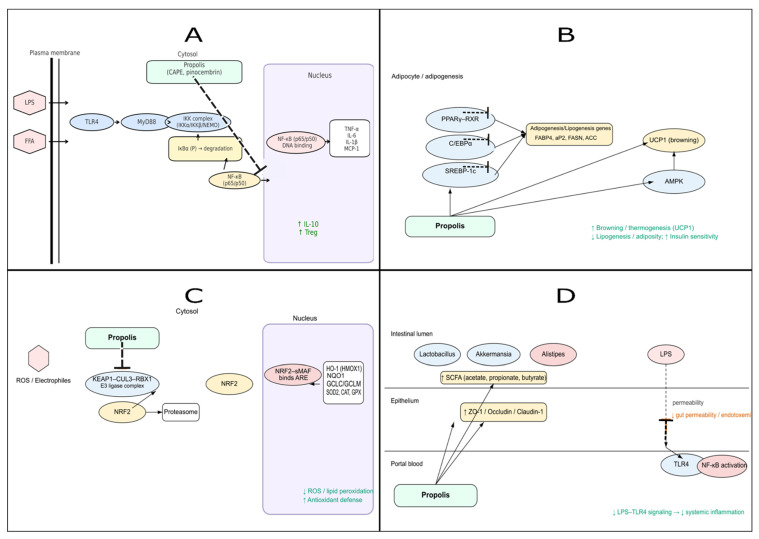
Proposed mechanistic pathways through which propolis may modulate obesity-related metabolic dysfunctions. (**A**) Modulation of TLR4/NF-κB inflammatory signaling pathway; (**B**) Regulation of adipogenesis, lipogenesis, browning, and AMPK activation; (**C**) Activation of Nrf2-mediated antioxidant defense mechanisms; (**D**) Modulation of gut microbiota composition, intestinal barrier integrity, and LPS-TLR4 signaling. The schematic was constructed using information from KEGG and Reactome pathway databases. Solid arrows indicate canonical signaling relationships, whereas dashed arrows represent proposed propolis-associated effects supported predominantly by preclinical evidence; therefore, causal inference in humans should be interpreted cautiously. The schematic was conceptualized and designed by the authors using Inkscape (version 1.4.3).

Propolis, through its diverse biological activities, may modulate multiple interconnected pathways implicated in the development and progression of obesity [60,61,62]. Obesity is marked by complex metabolic, hormonal, and inflammatory disturbances that disrupt energy balance. Key molecular mediators include adipocyte-derived hormones such as leptin and adiponectin, together with pro-inflammatory cytokines such as IL-6 and TNF-α, which collectively regulate glucose homeostasis, insulin sensitivity, adipocyte differentiation, and lipid storage [63]. Furthermore, imbalances in appetite-regulating hormones—notably leptin and ghrelin—are strongly associated with obesity-related metabolic dysfunctions [64]. Evidence from recent in vitro and in vivo studies suggests that propolis and its polyphenolic constituents may modulate these molecular targets through several mechanisms, including activation of antioxidant defenses via the nuclear factor erythroid 2–related factor 2 (Nrf2) signaling pathway, suppression of inflammatory signaling through nuclear factor kappa B (NF-κB) inhibition, regulation of adipogenesis and lipid metabolism, and modulation of gut microbiota composition [61,65,66,67,68]. The following subsections provide a detailed examination of these mechanisms, supported by experimental data. An integrated schematic overview of the proposed pathways is presented in Figure 2.

(A). NF-κB–mediated inflammatory signaling: Propolis is proposed to attenuate TLR4/MyD88-dependent NF-κB activation, which may reduce downstream expression of pro-inflammatory cytokines (TNF-α, IL-6, IL-1β, MCP-1) and may promote anti-inflammatory mediators (e.g., upregulation of IL-10 and Treg).
(B). Adipogenesis and lipid metabolism: Propolis may modulate transcriptional regulators of adipocyte differentiation (PPARγ–RXR, C/EBPα, SREBP-1c) and downstream adipogenic/lipogenic genes (FABP4, aP2, FASN, ACC), while potentially activating AMPK and UCP1. Collectively, these effects may contribute to enhanced browning/thermogenesis, reduced lipogenesis, and improved insulin sensitivity.
(C). Nrf2-driven antioxidant response: Propolis may interfere with KEAP1-mediated degradation of Nrf2, thereby facilitating nuclear translocation and activation of ARE-dependent antioxidant genes (HO-1, NQO1, SOD, CAT, GPx). These mechanisms may reduce ROS and lipid peroxidation and support restoration of redox balance.
(D). Gut microbiota and barrier integrity: Propolis may promote beneficial microbes (e.g., *Lactobacillus*, *Akkermansia*) and short-chain fatty acid production, while potentially reducing inflammation-associated taxa (e.g., *Alistipes*). These changes may enhance epithelial barrier proteins (ZO-1, Occludin, Claudin-1), reduce intestinal permeability/endotoxemia, and attenuate systemic inflammation via downregulation of LPS–TLR4 signaling.

Abbreviations: TLR4, Toll-like receptor 4; MyD88, myeloid differentiation primary response 88; AMPK, AMP-activated protein kinase; UCP1, uncoupling protein 1; KEAP1, Kelch-like ECH-associated protein 1; ARE, antioxidant response element; HO-1, heme oxygenase-1; NQO1, NAD(P)H quinone dehydrogenase 1; ZO-1, zonula occludens-1; Treg, regulatory T cell; RXR, retinoid X receptor; C/EBPα, CCAAT/enhancer-binding protein α; SREBP-1c, sterol regulatory element–binding protein 1c; FABP4, fatty acid-binding protein 4; FASN, fatty acid synthase; ACC, acetyl-CoA carboxylase.

### 4.1. Antioxidant Mechanisms and Oxidative Stress Regulation

Oxidative stress is a key driver of obesity-associated metabolic dysfunction. Chronic low-grade inflammation, hyperglycemia, elevated circulating free fatty acids, and impaired mitochondrial function collectively increase reactive oxygen species (ROS) production and disrupt redox homeostasis [69,70,71]. Persistent oxidative stress amplifies inflammatory signaling, impairs adipose tissue endocrine function, and aggravates insulin resistance; therefore, restoring redox balance has become an important therapeutic target in obesity research [72,73].

Propolis is a complex bee-derived matrix enriched in polyphenols and flavonoids with well-established antioxidant properties. Major constituents such as caffeic acid phenethyl ester (CAPE), chrysin, pinocembrin, galangin, and caffeic acid derivatives have been widely investigated for their ability to scavenge ROS and reduce lipid peroxidation, while also activating endogenous antioxidant defense systems [74,75,76,77]. Importantly, beyond direct radical-quenching activity, propolis appears to modulate redox-sensitive signaling networks that are closely linked to mitochondrial function and metabolic regulation.

#### 4.1.1. In Vitro Findings

Cell-based studies suggest that propolis extracts and selected constituents can reduce intracellular ROS accumulation and may protect against oxidative damage under certain experimental conditions [74]. A central mechanistic theme is the activation of the Nrf2 pathway, which regulates transcription of antioxidant and detoxifying enzymes via AREs [61,78,79]. In several in vitro models, propolis-derived polyphenols have been reported to increase Nrf2 nuclear translocation and upregulate downstream antioxidant enzymes, including HO-1, SOD, CAT, and glutathione-related enzymes, suggesting that propolis may enhance cellular antioxidant capacity [78,79].

In addition, propolis has been linked to improved mitochondrial performance under oxidative stress conditions. Experimental systems have reported improvements in mitochondrial membrane potential, intracellular antioxidant status, and oxygen utilization following exposure to propolis extracts or polyphenol-rich fractions [80,81,82]. Notably, these effects have been described using chemically diverse preparations, including ethanolic extracts and polyphenol-rich fractions, typically tested across a wide concentration range (often spanning ~1–100 µg/mL depending on the assay and cell type), which may partly explain variability in the magnitude of responses across studies [81,82]. Although these studies vary substantially in extract type, concentration range, and chemical characterization, they collectively suggest that propolis may stabilize mitochondrial redox balance at the cellular level.

Red propolis has also demonstrated mitochondria-related effects in cell-based systems. When tested at concentrations ranging from 10 to 80 µg/mL in antioxidant assays and 3.9 to 500 µg/mL in functional assays, red propolis extracts were reported to enhance mitochondrial activity, increase intracellular antioxidant capacity, and support the function of metabolically active tissues [83,84]. However, because many studies do not provide full chemical profiling, polyphenol standardization, or batch-to-batch quality control, these findings remain difficult to synthesize quantitatively. Such variability likely contributes to inconsistencies in reported outcomes.

Overall, in vitro evidence suggests antioxidant and mitochondria-protective actions of propolis largely through Nrf2-related mechanisms. However, heterogeneity in propolis preparation (ethanolic vs. other extracts), lack of consistent standardization, and wide dosing ranges limit direct cross-study comparability and translation.

#### 4.1.2. In Vivo Findings

Animal studies provide stronger preclinical support suggesting that propolis may improve systemic redox status in obesity-related contexts. In high-fat diet models, supplementation with polyphenol-rich ethanolic propolis preparations—including dried ethanolic extract powders such as Propolis Extract Powder (PEP) and Poplar Propolis Ethanolic Extract (PPEE) powder—has been associated with improvements in glucose tolerance, lipid oxidation and thermogenesis, and reduced expression of inflammatory mediators, with these outcomes mechanistically linked to Nrf2 activation in metabolic tissues [85,86]. These products are typically obtained by ethanolic extraction followed by solvent removal and drying, yielding powder formulations that facilitate dosing consistency in long-term feeding studies. In a high-fat diet-induced obesity model, daily supplementation with a polyphenol-rich ethanolic propolis preparation at a dose of 20 mg per mouse (equivalent to ~4.5 mg total polyphenols) for 12 weeks was associated with improvements in glucose tolerance and markers of lipid oxidation and thermogenesis, alongside lower expression of inflammatory genes, consistent with Nrf2 activation [85].

In parallel, mitochondrial dysfunction—characterized by impaired oxidative phosphorylation, reduced energy production, and elevated ROS—is a hallmark of obesity-related metabolic disease [69]. In vivo findings suggest that propolis may counteract these alterations by supporting mitochondrial biogenesis and preserving mitochondrial integrity [87,88]. Nevertheless, these outcomes should be interpreted as preclinical efficacy signals rather than definitive evidence of causality in humans.

While CAPE is frequently discussed as a key bioactive molecule, its mechanistic evidence derives largely from experimental models beyond obesity. For example, in a murine spinal cord injury model, intraperitoneal CAPE administration activated the SIRT1/PGC-1α axis and reduced mitochondrial fragmentation through DRP1-related signaling [89]. These data provide mechanistic plausibility for CAPE-mediated mitochondrial protection; however, they should be interpreted as supportive evidence rather than direct confirmation of obesity-specific mechanisms. Obesity-specific animal studies are still needed to confirm whether comparable pathways operate in adipose tissue, liver, and skeletal muscle under metabolic stress.

Preclinical evidence suggests that propolis may improve oxidative balance and mitochondrial function in obesity-related models, with Nrf2 signaling and mitochondrial biogenesis emerging as recurrent mechanistic themes. However, interpretation is constrained by heterogeneity in propolis source, extraction method, and standardization, and by limited mechanistic confirmation in target metabolic tissues.

#### 4.1.3. Clinical Findings

Clinical data remain limited but are broadly consistent with the concept that propolis may improve redox and inflammatory status in metabolic disease settings. Across human trials, propolis supplementation has been associated with improvements in systemic antioxidant capacity markers and reductions in selected oxidative stress indicators, often alongside modest improvements in metabolic parameters [90,91]. In clinical studies, propolis has been administered in heterogeneous formats, including powders, commercially available market products, and capsule-based supplements, often with limited reporting of botanical origin, extraction method, and polyphenol/CAPE content [92]. However, the clinical literature is characterized by variability in dose, duration, population (e.g., obesity vs. obesity-related comorbidities), and—critically—the chemical characterization of the administered propolis product. In most studies, the polyphenol profile, CAPE content, and standardization procedures are insufficiently reported, limiting mechanistic interpretation and reproducibility [33,93,94].

Human evidence suggests potential benefits of propolis on systemic oxidative stress markers, but conclusions remain preliminary due to heterogeneous trial designs, limited standardization of preparations, and inconsistent reporting of polyphenol content and primary endpoints.

Collectively, available evidence suggests that propolis may target multiple nodes in obesity-associated oxidative stress, including Nrf2-dependent antioxidant defenses, mitochondrial biogenesis and function, and ROS-generating enzymes such as NADPH oxidase. These mechanisms may interact to preserve metabolic homeostasis by reducing oxidative damage, limiting inflammatory amplification, and supporting insulin-sensitive energy metabolism. Importantly, most mechanistic links summarized here are derived from in vitro systems, animal models, or isolated-compound experiments; thus, mechanistic causality in humans remains to be confirmed. Figure 2 summarizes these pathways and highlights mechanistic links supported predominantly by preclinical evidence [61,77,83].

### 4.2. Anti-Inflammatory Mechanisms and Immune Modulation

Chronic low-grade inflammation is a defining feature of obesity and plays a central role in the development of insulin resistance, type 2 diabetes, non-alcoholic fatty liver disease, and cardiovascular complications [95,96]. As adipose tissue expands, it undergoes structural and immunological changes, including adipocyte hypertrophy, local hypoxia, and recruitment of pro-inflammatory immune cells—particularly M1 macrophages. These changes drive increased secretion of inflammatory mediators such as TNF-α, IL-6, and CRP, which propagate systemic inflammation [97,98,99]. Elevated leptin levels under hypoxic and inflammatory conditions further contribute to this cycle by promoting leptin resistance and worsening metabolic dysfunction [100,101,102,103]. Because of these mechanisms, signaling pathways involving NF-κB, IL-6, and TNF-α are increasingly targeted in therapeutic strategies. Propolis and its bioactive polyphenols—such as artepillin C, kaempferide, and baccharin—have emerged as promising natural modulators of these inflammatory cascades [12,13].

Macrophage-based in vitro systems have provided mechanistic insights into the anti-inflammatory potential of propolis constituents, particularly through suppression of inflammatory mediator production. In RAW 264.7 macrophages, exposure to 1–100 μg/mL for 20 h inhibited NO synthesis [104]. These findings support the hypothesis that propolis-derived polyphenols may attenuate inflammatory signaling at the cellular level, including pathways upstream of NF-κB activation.

#### 4.2.1. In Vitro Findings

In vitro studies suggest that propolis and selected constituents can suppress inflammatory mediator production in immune cell models [14,105]. However, these systems do not fully capture the complexity of obesity-associated metaflammation, and dose ranges, extract composition, and standardization vary widely across experiments [93,94].

#### 4.2.2. In Vivo Findings

Animal studies provide complementary insights. High-fat diet feeding induces chronic inflammation in adipose tissue and contributes to systemic metabolic dysregulation, a process often termed “metaflammation” [106,107]. Propolis extracts have been reported to attenuate these responses by suppressing pro-inflammatory gene expression, lowering nitric oxide (NO) production, and reducing cytokine levels—including TNF-α and IL-6—through downregulation of NF-κB signaling [108]. Artepillin C may contribute to the reported anti-inflammatory activity. In addition to obesity-relevant chronic models, several studies have used acute inflammation assays (e.g., carrageenan-induced edema) or macrophage-based in vitro systems to demonstrate the anti-inflammatory potential of propolis constituents, although these models do not fully recapitulate obesity-associated metaflammation [14]. In a carrageenan-induced paw edema model, intraperitoneal injection of 1–10 mg/kg artepillin C, administered 30 min prior to the challenge, significantly lowered prostaglandin E2 and neutrophil infiltration [47]. Oral administration of Tigzirt propolis as an ethyl acetate extract of propolis (EAP; dry extract) at 50–250 mg/kg/day for five days in the same model further reduced TNF-α, prostaglandin E2 (PGE2), myeloperoxidase (MPO), and malondialdehyde (MDA) levels [109].

Additional models support these anti-inflammatory actions. Experiments in both obese and lean mouse models have shown that repeated intraperitoneal injections of ethanolic propolis extract at 100 mg per kilogram, administered twice weekly for four weeks, shifted immune responses toward an anti-inflammatory profile as demonstrated by Kitamura et al. [66]. In line with these observations, Hsieh et al. reported that giving Taiwanese green propolis extract preparation orally at a dose of 20 mg per kilogram at 0, 24, and 48 h in a monosodium urate–induced peritonitis model reduced neutrophil infiltration and lowered cytokine production [110]. Together, these findings suggest that propolis may modulate inflammation through multiple converging mechanisms—acting both at the level of inflammatory mediator production and immune cell polarization. These findings support broader immunomodulatory potential, while obesity-associated metaflammation is best interpreted based on diet-induced obesity and metabolically relevant models. Figure 2 provides a schematic overview of these mechanisms.

Preclinical evidence suggests that propolis preparations and selected constituents may attenuate inflammatory signaling and cytokine production, partly through modulation of NF-κB-related pathways and immune cell polarization. However, interpretation is limited by the use of heterogeneous models (diet-induced obesity vs. acute inflammation), variable dosing regimens, and incomplete standardization of propolis preparations.

#### 4.2.3. Clinical Findings

Clinical evidence, though limited, is broadly consistent with these observations. A 2020 meta-analysis that pooled data from six randomized controlled trials reported significant reductions in circulating TNF-α and CRP following propolis supplementation [111]. In a triple-blind, placebo-controlled trial with 60 women diagnosed with polycystic ovary syndrome, daily supplementation with 500 mg of standardized propolis (containing 90 mg polyphenols and 67 mg flavonoids) for 12 weeks led to a measurable reduction in high-sensitivity C-reactive protein (hs-CRP) concentrations, without any adverse events [112]. In a separate study in patients with non-alcoholic fatty liver disease, four months of supplementation with 500 mg/day of Iranian poplar propolis administered as a tablet containing a lyophilized ethanolic extract improved hepatic steatosis and fibrosis, accompanied by a decrease in hs-CRP levels [113]. Taken together, these clinical data, although limited in scope, suggest a potential anti-inflammatory effect of propolis in metabolic disorders. Most studies are small and vary in duration and formulation, making it difficult to draw firm conclusions. More standardized and larger clinical trials are needed to confirm these effects.

Human trials and meta-analytic evidence suggest that propolis supplementation may reduce selected systemic inflammatory markers (e.g., TNF-α, CRP/hs-CRP). Nevertheless, the clinical literature remains limited by small sample sizes, heterogeneous populations, and variability in formulation and standardization, preventing strong causal inference regarding obesity-associated inflammation.

Collectively, available evidence suggests that propolis preparations and selected constituents may attenuate obesity-associated inflammatory signaling, particularly through modulation of NF-κB-related pathways, reduced pro-inflammatory cytokine production, and shifts in immune cell polarization. However, given the heterogeneity in experimental models and propolis standardization, and the limited size and duration of clinical trials, mechanistic causality in humans remains to be confirmed (Figure 2).

### 4.3. Adipogenesis and Lipid Metabolism Homeostasis

Adipogenesis involves a series of transcriptional events through which precursor cells differentiate into mature adipocytes [114]. The key regulators are members of the CCAAT/enhancer-binding protein (C/EBP) family (C/EBPβ, C/EBPδ, C/EBPα) and peroxisome proliferator-activated receptor gamma (PPARγ), the master regulator of adipocyte differentiation [115]. Additional regulatory elements—such as Krüppel-like factors (KLFs), components of the Wnt signaling pathway, and cell-cycle modulators—further refine adipogenic commitment and maturation [116,117,118]. Because adipogenesis plays a central role in energy storage and endocrine balance, it remains a major area of focus in obesity research [119].

#### 4.3.1. In Vitro Findings

Mechanistic and cell-based studies provide insight into how propolis-derived phenolics may influence adipogenic signaling and lipid metabolic programs [120,121]. Phenolic compounds such as artepillin C, kaempferide, and isokaempferide have been reported to activate the free fatty acid receptor 4 (FFAR4) pathway, which is linked to enhanced GLUT4 translocation, increased glucose uptake, and modulation of adipocyte differentiation–related signaling. In HEK293 cells expressing FFAR4, chrysin, pinocembrin, galangin, pinobanksin, and CAPE similarly activated FFAR4 after short-term exposure at 0.1% dimethyl sulfoxide (DMSO) [120]. 2-phenyl-8-(1-phenylallyl)-chromenone has been described as a pan-PPAR modulator, engaging PPARα, PPARβ/δ, and PPARγ to influence transcriptional programs related to fatty acid oxidation and adipogenesis; short-term exposure (1–2 h) in human mesenchymal stem cells and HEK293 assays was associated with reductions in intracellular triglyceride levels [122]. Flavonoids such as tectochrysin and artepillin C have also been identified as RXR or PPARγ ligands in HEK293 luciferase and 3T3-L1 assays [123]. Importantly, adipogenesis is a stage-dependent and tissue-specific process, and activation of FFAR4 or RXR/PPARγ signaling in cell-based assays may reflect adipocyte metabolic programming rather than uniform promotion of fat-mass expansion. These findings highlight the complex and context-dependent actions of propolis constituents on adipogenic pathways.

In vitro studies suggest that propolis-derived constituents can interact with FFAR4 and nuclear receptor signaling (RXR/PPAR), which may influence adipogenic and lipid metabolic gene programs. However, these mechanistic findings are derived from heterogeneous assay systems, short-term exposures, and isolated-compound experiments; therefore, their physiological relevance to obesity-related adipose tissue remodeling requires confirmation in metabolically relevant models.

#### 4.3.2. In Vivo Findings

Animal studies provide preclinical evidence that propolis preparations may modulate lipid metabolism and adipose tissue remodeling, although the magnitude and mechanistic drivers vary by model and preparation [124,125]. In male C57BL/6 mice, daily oral administration of Brazilian propolis as a powdered ethanolic extract at 200 mg/kg for five weeks was associated with improved glycemic regulation and lower circulating triglyceride and cholesterol concentrations, with proposed involvement of RXRα-related regulation of lipid uptake and oxidation genes [126]. Longer-term administration in SAMP8 mice (200 mg/kg/day for 12 weeks) reduced hepatic triglyceride accumulation and improved serum lipid profiles [127]. Similar lipid-lowering trends were also reported in Wistar rats fed high-fat diets with propolis mixed directly into the diet (e.g., supplemented with 0.05% propolis, *w*/*w*; 0.05–0.5% range) for eight weeks, and in C57BL/6 mice receiving Chinese propolis extracts at 30–60 mg/kg/day for one week in a Triton-induced hyperlipidemia model [128].

Some studies have specifically focused on adipose tissue thermogenic remodeling. In obesity models, Taiwanese green propolis administered as a dietary supplement at 500 or 1000 ppm for 12 weeks was reported to promote browning of white adipose tissue by upregulating UCP1 [124]. Importantly, induction of UCP1-mediated browning reflects thermogenic remodeling of mature adipocytes, whereas downregulation of PPARγ/C/EBPα primarily relates to inhibition of adipogenic differentiation and lipid storage programs. Likewise, caffeic acid phenethyl ester (CAPE) provided in the diet at 0.02–0.5% (*w*/*w*) for five weeks was reported to downregulate PPARγ and C/EBPα and inhibit MAPK signaling, consistent with suppression of adipogenic differentiation [129].

Preclinical studies suggest that propolis preparations (e.g., Brazilian, Chinese, and Taiwanese green propolis) and CAPE may be associated with improvements in circulating and hepatic lipid parameters and with adipose tissue remodeling (including browning-related markers). Nevertheless, interpretation is limited by heterogeneity in botanical origin, extraction procedures, and standardization of propolis preparations, as well as differences in model type, intervention duration, and dosing strategy.

#### 4.3.3. Clinical Findings

Clinical findings provide limited but supportive evidence that propolis supplementation may influence lipid metabolism in humans, although study populations and preparations are heterogeneous [130]. In a randomized controlled trial, daily administration of a 3% Beepolis^®^ solution (Laboratorio Rotterdam Ltda, Maule Region, Chile) (15 drops twice daily for 90 days) increased HDL-cholesterol levels and reduced thiobarbituric acid reactive substances (TBARSs), suggesting changes in lipid-related and oxidative status markers [131]. In patients with type 2 diabetes, oral supplementation with propolis administered as capsule-based supplements (500 mg twice daily for three months) improved lipid parameters, potentially reflecting changes in hepatic lipid regulation and antioxidant activity [132]. Similar outcomes were reported in women with rheumatoid arthritis, where 12 weeks of propolis supplementation (500 mg capsules twice daily containing 170 mg poplar-type propolis ethanolic extract, standardized to 100 mg polyphenols and 67 mg flavonoids per capsule) lowered total cholesterol, LDL, and triglycerides, while increasing HDL [133]. Taken together, available clinical data are broadly consistent with the possibility that propolis supplementation may influence lipid homeostasis.

Human trials suggest that propolis supplementation (administered as solutions and capsule-based supplements) may be associated with modest improvements in lipid parameters and oxidative stress markers. However, clinical interpretation is constrained by heterogeneity in participant populations (metabolic disease vs. inflammatory conditions), variable formulations, and limited reporting of botanical origin, extraction method, and polyphenol standardization.

**Figure 3:**
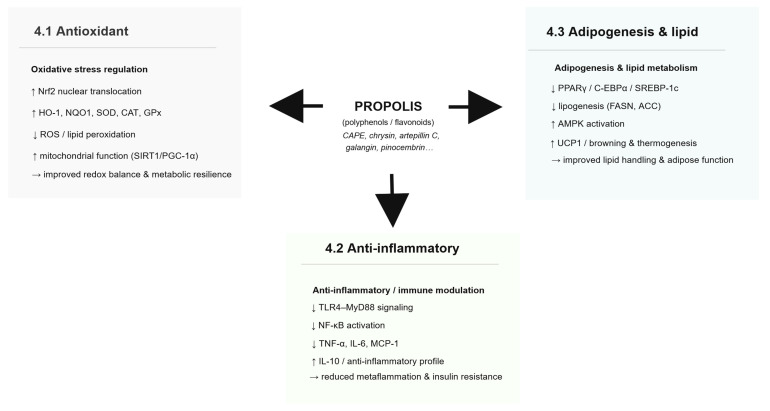
Simplified summary of the core mechanisms through which propolis may modulate obesity-related metabolic dysfunctions (Section 4.1, Section 4.2 and Section 4.3). Arrows indicate the directional relationship between propolis and the major mechanistic domains (antioxidant, anti-inflammatory, and adipogenesis/lipid metabolism pathways). **Abbreviations:** CAPE, caffeic acid phenethyl ester; Nrf2, nuclear factor erythroid 2–related factor 2; HO-1, heme oxygenase-1; NQO1, NAD(P)H quinone dehydrogenase 1; SOD, superoxide dismutase; CAT, catalase; GPx, glutathione peroxidase; TLR4, Toll-like receptor 4; MyD88, myeloid differentiation primary response 88; NF-κB, nuclear factor kappa B; AMPK, AMP-activated protein kinase; UCP1, uncoupling protein 1. The schematic was conceptualized and designed by the authors using Inkscape (version 1.4.3).

Collectively, evidence from mechanistic assays, animal models, and a limited number of clinical trials suggests that propolis preparations and selected constituents may influence adipogenesis-related signaling and lipid metabolism homeostasis through multiple pathways, including FFAR4 and RXR/PPAR-related mechanisms, adipogenic transcriptional regulation, and thermogenic remodeling. However, most mechanistic links remain derived from in vitro systems, animal studies, or isolated-compound experiments; therefore, causal inference and mechanistic confirmation in humans remain to be established. A simplified graphical summary of the key mechanisms discussed in Section 4.1, Section 4.2 and Section 4.3 is provided in Figure 3.

Emerging evidence suggests that the gut microbiota may play an important role in metabolic inflammation and energy homeostasis in obesity. Obesity-associated dysbiosis is frequently characterized by reduced short-chain fatty acid (SCFA) production, impaired intestinal barrier integrity, and increased abundance of lipopolysaccharide (LPS)-producing taxa, which together may promote endotoxemia and activation of inflammatory signaling pathways such as TLR4/NF-κB [134,135]. Propolis has recently gained attention as a microbiota-targeting agent because its complex polyphenolic matrix can exert both prebiotic-like and antimicrobial activities, thereby potentially influencing microbial composition and downstream host pathways relevant to metabolic inflammation [136,137]. Importantly, the magnitude and direction of these effects may depend on propolis type, botanical origin, extraction method, and degree of standardization [138].

### 4.4. Gut-Microbiota Interactions 

Growing experimental evidence indicates that propolis may modulate gut microbiota composition and intestinal barrier integrity in metabolically relevant settings, with reported effects on SCFA production, tight junction function, and inflammatory signaling pathways [139,140,141,142,143,144,145,146,147,148,149].

#### 4.4.1. In Vitro Findings

In vitro fermentation studies provide complementary findings suggesting a prebiotic-like role of propolis [139,140]. Fermentation of fecal samples from healthy adults and IBD patients with 0.5 mg/mL standardized poplar-type propolis for 48 h increased SCFA production and promoted beneficial microbial shifts, indicating that propolis-derived constituents may modulate microbial metabolism under controlled conditions [140].

In vitro fermentation data suggest that standardized propolis preparations may increase SCFA output and promote favorable microbial shifts. However, these findings are derived from ex vivo systems and do not directly capture the complexity of host–microbiota interactions, barrier function, and metabolic inflammation in obesity.

#### 4.4.2. In Vivo Findings

Preclinical models provide broader findings suggesting that propolis may influence microbiota composition and intestinal barrier function in ways that are mechanistically relevant to obesity-associated metabolic inflammation [141,142,143,144]. Across diverse rodent studies, a recurring pattern is enrichment of SCFA-producing taxa alongside reductions in putative LPS-associated genera, accompanied by improvements in tight junction integrity and attenuation of inflammatory signaling [144].

Several studies suggest that propolis may support SCFA-associated metabolic regulation [139,145]. In db/db mice, dietary supplementation with 0.08–2% Brazilian green propolis for eight weeks enriched SCFA-producing genera such as *Butyricicoccus* and *Acetivibrio*, strengthened mucosal barrier function, and was associated with improvements in glucose intolerance and systemic inflammation [146]. Similarly, in high-fat diet-fed mice, 12-week supplementation with 1–2% Chinese propolis extract increased *Lactobacillus* abundance, reduced LPS-producing taxa such as *Alistipes*, and improved insulin sensitivity [147]. In a metabolically distinct model, male ICR mice receiving 100–200 mg/kg ethanolic propolis extract for four weeks showed increased *Akkermansia* and *Lactobacillus*, elevated SCFA production, and reduced oxidative stress [148], supporting the plausibility that propolis can modulate SCFA-linked microbial outputs.

In parallel, multiple studies indicate that propolis may preserve or restore intestinal barrier integrity, which is a key upstream determinant of endotoxemia-driven metaflammation. In rats exposed to aflatoxin B1, daily oral gavage of 250 mg/kg propolis for four weeks restored tight junction integrity, reduced oxidative stress, and maintained beneficial taxa, including *Lactobacillus*, *Roseburia*, and *Phascolarctobacterium* [149]. Likewise, daily oral gavage of 80–240 mg/kg propolis for four weeks in diabetic rodents upregulated tight junction proteins, increased SCFA levels, and improved glycemic control while reducing inflammatory markers [144]. In high-fat diet (HFD)/streptozotocin (STZ)-induced diabetic mice, administration of 600 mg/kg ethanolic propolis extract for four weeks lowered blood glucose and pro-inflammatory cytokines, increased beneficial genera (*Lactobacillus, Akkermansia, Parabacteroides*), and decreased *Bilophila* and *Desulfovibrio*; these changes were interpreted as potentially contributing to improved insulin sensitivity [143].

Notably, propolis has also been reported to modulate upstream inflammatory triggers such as TLR4 signaling. In a DSS-induced colitis model, rats fed a Western-style diet with 0.3% Chinese propolis for 21 days showed reduced disease severity, increased *Lactobacillus* and *Bifidobacterium*, reduced *Escherichia–Shigella*, and inhibition of TLR4/NF-κB signaling [142]. Although colitis is not an obesity model, these findings remain mechanistically relevant because barrier disruption and TLR4-driven inflammatory amplification represent shared upstream nodes linking gut dysfunction to systemic metabolic inflammation.

Across rodent models, propolis preparations (Chinese propolis, Brazilian green propolis, and ethanolic extracts) have been reported to (i) enrich SCFA-producing taxa, (ii) reduce putative LPS-associated genera, (iii) strengthen tight junction integrity, and (iv) attenuate inflammatory signaling such as TLR4/NF-κB. Nevertheless, interpretation is limited by heterogeneity in model systems (obesity, diabetes, colitis, toxin exposure), variability in propolis type and extraction method, and incomplete chemical standardization, which complicate cross-study comparability. However, many studies provide limited chemical characterization (e.g., total polyphenols, CAPE content), which restricts reproducibility.

#### 4.4.3. Clinical Findings

Human evidence remains limited. In a double-blind, placebo-controlled trial with 42 hemodialysis patients, eight weeks of daily green propolis extract (400 mg) slightly increased gut microbial diversity, and inverse correlations were reported between uremic toxin levels and several bacterial groups, including Firmicutes, Lentisphaerae, and Proteobacteria [150]. Although this cohort does not represent obesity, the trial provides preliminary human evidence that propolis supplementation may influence gut microbial ecology, which is mechanistically relevant to metabolic inflammation [139,151].

Available clinical evidence suggests that propolis supplementation may influence gut microbial diversity in humans; however, obesity-specific randomized trials linking microbiota changes to metabolic endpoints remain scarce. In addition, limited reporting of propolis composition and polyphenol standardization restricts mechanistic interpretation and reproducibility.

**Figure 4:**
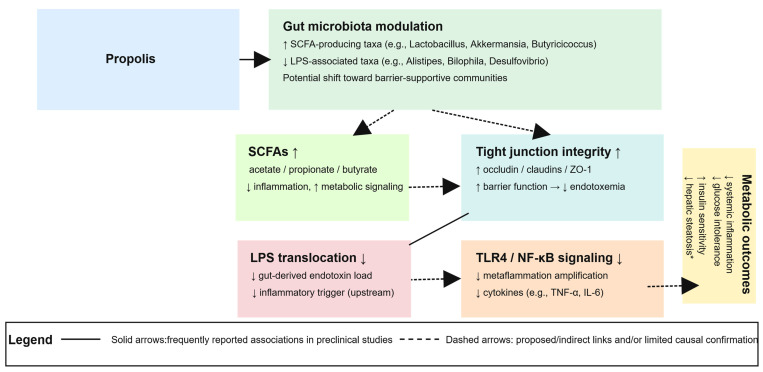
Proposed gut microbiota–related mechanisms through which propolis may influence obesity-associated metabolic inflammation. The schematic was conceptualized and designed by the authors using Inkscape (version 1.4.3). Abbreviations: SCFA, short-chain fatty acid; LPS, lipopolysaccharide; TLR4, Toll-like receptor 4; NF-κB, nuclear factor kappa B. The asterisk indicates that the association with hepatic steatosis is supported by relatively limited or indirect evidence compared with other metabolic outcomes shown in the schematic.

Collectively, available evidence suggests that propolis preparations may influence gut microbiota composition and intestinal barrier function through mechanisms relevant to obesity-associated metaflammation, particularly via modulation of SCFA production, tight junction integrity, and LPS/TLR4-driven inflammatory signaling. However, most mechanistic links remain derived from preclinical models and heterogeneous disease contexts; therefore, causal inference and mechanistic confirmation in obesity-focused human trials remain to be established. A schematic summary of these proposed gut microbiota–related pathways is provided in Figure 4.

An integrated overview linking the mechanisms discussed in this section and highlighting convergent pathways is provided in Figure 2, Figure 3 and Figure 4.

## 5. Propolis in Obesity-Linked Chronic Diseases: Evidence from Experimental and Clinical Studies

**Figure 5:**
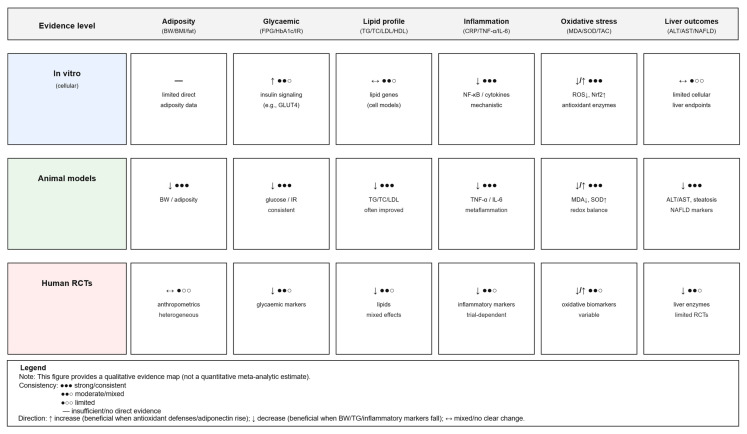
Evidence-level graphical summary of propolis effects across mechanistic, preclinical, and clinical studies. The schematic was conceptualized and designed by the authors using Inkscape (version 1.4.3). Abbreviations: ALT, alanine aminotransferase; AST, aspartate aminotransferase; BMI, body mass index; BW, body weight; CRP, C-reactive protein; FPG, fasting plasma glucose; HbA1c, glycated hemoglobin; HDL, high-density lipoprotein; IL-6, interleukin-6; IR, insulin resistance; LDL, low-density lipoprotein; MDA, malondialdehyde; NAFLD, non-alcoholic fatty liver disease; RCT, randomized controlled trial; SOD, superoxide dismutase; TAC, total antioxidant capacity; TC, total cholesterol; TG, triglycerides; TNF-α, tumor necrosis factor-α.

To provide a comprehensive overview of the evidence base, this section presents both clinical and experimental studies examining the effects of propolis in obesity-related metabolic disorders. Table 2 summarizes key randomized controlled trials (RCTs) reporting on metabolic, inflammatory, and anthropometric outcomes, while Table 3 and Table 4 outline findings from experimental animal models that investigate the mechanistic and physiological effects of propolis across obesity, diabetes, metabolic syndrome, and related conditions. To provide an integrated qualitative overview of the available evidence across mechanistic, preclinical, and clinical levels, a graphical evidence map is presented in Figure 5.

### 5.1. Obesity

Evidence regarding the potential anti-obesity effects of propolis spans in vitro cellular systems, experimental animal models, and human clinical trials [62,174,175]. At the cellular level, in vitro treatment of 3T3-L1 preadipocytes with Taiwanese green propolis ethanol extract (1–5 μg/mL) promoted adipocyte differentiation and reversed TNF-α-mediated suppression of adiponectin expression, suggesting a potential role in preserving adipose tissue function and insulin sensitivity [62]. Notably, differentiation-related effects observed in 3T3-L1 assays may reflect restoration of adipocyte function and insulin sensitivity under inflammatory stress rather than promotion of adipose expansion in vivo. Taken together, these findings suggest that propolis may influence obesity-related pathways through complementary metabolic, hormonal, and inflammatory mechanisms.

Experimental animal models have frequently reported anti-obesity–related effects of propolis across different preparations and doses. In mice fed a high-fat diet, oral administration of propolis at doses between 2.5 and 25 mg per kilogram of body weight for four weeks reduced body weight gain, visceral adiposity, and hepatic lipogenic gene expression, while improving lipid profiles [176]. Similarly, supplementing the diet of C57BL/6J mice with 1–2% ethanolic propolis extract over a 12-week period was associated with reduced weight gain and adiposity and improved glucose tolerance, lipid metabolism, and gut microbiota composition [174]. Further evidence from C57BL/6J mice indicates that a 14-week supplementation with 2% Brazilian green propolis decreased epididymal and subcutaneous fat mass and reduced immune cell infiltration in adipose tissue, likely through decreased lipid absorption and increased fecal lipid loss [177]. Intraperitoneal administration of Brazilian green propolis at 100 mg per kilogram for one month suppressed food intake and weight gain by upregulating leptin gene expression in adipose tissue, though this effect was absent in leptin-deficient ob/ob mice [178].

Comparable effects have been observed in rat models. In Wistar rats previously maintained on a palmitic acid-rich high-fat diet, nine weeks of daily Geniotrigona thoracica propolis extract at 300 mg per kilogram reduced weight gain, lowered neuroinflammatory cytokines such as IL-1β and TNF-α, and modulated autophagy-related proteins in the prefrontal cortex [179]. In standard diet-fed Wistar rats, 90 days of supplementation with 2% natural propolis decreased fat mass while increasing lean mass, insulin, and leptin levels, indicating improved body composition and endocrine function [180]. Methodological differences between these studies—including variations in propolis type, extraction method, dosage, intervention duration, and experimental models—should be considered when comparing outcomes, as they may partly explain inconsistencies across animal studies and mirror the heterogeneity seen in clinical research.

Current clinical findings suggest that propolis may be associated with changes in selected obesity-related parameters, including fat mass, body weight, and systemic inflammation [62,174,179,181]. A dose–response meta-analysis pooling 24 randomized controlled trials reported significant improvements in liver enzyme profiles with daily supplementation ranging from 100 to 1500 mg, although effects on body weight, body mass index (BMI), and adiposity were inconsistent [175]. A short-term trial in centrally obese adults found that 14 days of combined Trigona honey at 105 mg per day and propolis at 60 mg per day lowered serum leptin concentrations, suggesting favorable modulation of adipokine signaling [181]. Overall, the variability in observed outcomes appears to depend on factors such as dose, intervention duration, population characteristics, and extract type.

Taken together, the available clinical literature suggests that propolis supplementation may be associated with modest changes in selected obesity-related biomarkers (e.g., leptin and inflammatory mediators), whereas effects on body weight, BMI, and adiposity are not consistently observed across trials. In preclinical models, reductions in weight gain and fat accumulation are reported more frequently, often alongside improvements in lipid metabolism and inflammatory profiles; however, these findings should be interpreted cautiously due to differences in experimental design and dosing relative to humans. Importantly, cross-study comparison is limited by heterogeneity in propolis type, extraction procedures, and chemical standardization, as well as variability in intervention duration and population characteristics. Many clinical trials are also relatively short and may not be sufficiently powered for anthropometric outcomes, and dietary control is often limited. Therefore, current evidence does not support firm conclusions regarding weight-loss efficacy, but it does indicate a potential adjunctive role for propolis in metabolic and inflammatory modulation within obesity-related contexts.

### 5.2. Diabetes Mellitus

Diabetes mellitus is a progressive metabolic disorder characterized by chronic hyperglycemia due to impaired insulin secretion, insulin action, or both. As its global prevalence continues to rise, there is growing interest in safe and effective complementary strategies for glycemic regulation and the prevention of diabetes-related complications [182]. Clinical and preclinical evidence suggests that propolis may exert beneficial metabolic effects in diabetes through its antioxidant, metabolic, and immunomodulatory effects [183,184,185,186,187,188,189].

Cell-based studies reveal direct molecular mechanisms. Ethanolic propolis extracts strongly inhibited α-amylase, with an IC_50_ of 0.62 µg/mL, and α-glucosidase, with an IC_50_ of 40.40 µg/mL, and these effects varied depending on solvent and formulation [67,190]. These inhibitory activities persisted after simulated gastrointestinal digestion. In addition, propolis activated PPAR-γ in adipocytes, stimulated adiponectin secretion, and counteracted TNF-α-induced insulin resistance [191].

Animal studies provide mechanistic insight into these effects. In diabetic rodents, oral administration of propolis at doses between 200 and 300 mg per kilogram for one to ten weeks improved fasting glucose, lipid profiles, and antioxidant enzyme activity, including SOD, catalase, and GPx, while alleviating oxidative tissue damage [185]. Daily treatment with Turkish propolis water extract at 250 mg per kilogram for thirty-five days improved glycemia, HbA1c, lipid metabolism, and pancreatic and hepatic histology in STZ-induced rats [192]. In a type 1 diabetes model, Iranian propolis at doses of 100 to 200 mg per kilogram for six weeks reversed hyperglycemia, renal hypertrophy, and oxidative stress [188]. Taiwanese green propolis administered at 183.9 or 919.5 mg per kilogram for eight weeks preserved β-cell function and upregulated hepatic lipid metabolism genes such as PPAR-α and CYP7A1 [184]. Moroccan propolis at daily doses between 50 and 100 mg per kilogram for fifteen days improved glycemia, lipid profiles, and organ function, sometimes outperforming glibenclamide [193]. In ob/ob mice, intraperitoneal injection of Brazilian propolis at 100 mg per kilogram twice weekly for twelve weeks improved glycemia, cholesterol, insulin sensitivity, and immune balance in adipose tissue [194]. By contrast, short-term low-dose regimens of 10 or 90 mg per kilogram per day for seven days produced no significant effects, underscoring the importance of sufficient dose and duration [165].

Systematic reviews and meta-analyses suggest that propolis may represent a potential adjunct for glycemic control. Across animal studies, propolis was typically administered in doses ranging from 100 to 300 mg per kilogram of body weight, while human trials generally used daily doses of around 250 mg over periods of two to twelve weeks. Supplementation at these levels has been associated with significant reductions in fasting glucose and HbA1c, though effects on insulin and HOMA-IR have been more variable [91,189,190,192,193,194,195,196,197,198,199].

Clinical trials provide evidence for dose- and duration-dependent effects of propolis on glycemic regulation and oxidative stress. In patients with type 2 diabetes, daily supplementation with 1500 mg of Iranian propolis for eight weeks lowered fasting glucose, HOMA-IR, IL-6, and IL-17 while improving insulin sensitivity markers such as HOMA-B and QUICKI [197]. In individuals with insulin resistance, twelve weeks of standardized poplar propolis at 900 mg per day enhanced insulin sensitivity [198]. Supplementation with 1500 mg per day for eight weeks also improved total antioxidant capacity, SOD, and GPx levels, alongside reductions in fasting glucose, two-hour postprandial glucose, insulin, and HbA1c [183]. An eighteen-week intervention with 900 mg per day of Chinese propolis improved oxidative stress markers without significantly altering glycemic indices [186]. While these findings are promising, variability in sample sizes, intervention durations, and extract compositions across trials introduces methodological heterogeneity that should be considered when interpreting clinical evidence.

Overall, the literature indicates that propolis supplementation may contribute to improvements in glycemic indices in some clinical settings, particularly in type 2 diabetes, where reductions in fasting glucose and HbA1c have been reported in several trials and meta-analyses. These findings are often accompanied by favorable changes in oxidative stress markers and inflammatory mediators, which are consistent with mechanistic evidence from animal and cell-based studies. Nevertheless, the clinical evidence base remains heterogeneous, with variability in extract composition, polyphenol content, baseline glycemic control, and concurrent medication use, all of which may influence observed outcomes. In addition, intervention duration is frequently limited, and long-term sustainability of effects is uncertain. Accordingly, while the current evidence supports further investigation of propolis as a complementary approach in diabetes, well-powered randomized trials using chemically standardized preparations and clinically meaningful endpoints are still required before robust conclusions can be drawn.

### 5.3. Metabolic Syndrome

Recent research suggests that propolis may have potential as a complementary approach in metabolic syndrome, acting through multiple pathways that involve inflammation, lipid metabolism, insulin signaling, and gut microbiota regulation. These biological effects provide a mechanistic foundation for its potential clinical impact.

At the molecular level, in vitro assays demonstrated that Zuccagnia-type Argentine propolis strongly inhibited α-glucosidase, α-amylase, and lipase enzymes (IC_50_: 7–48 µg/mL) while exhibiting pronounced antioxidant activity [200]. These molecular effects complement the clinical and preclinical findings, reinforcing the multi-targeted actions of propolis on core mechanisms underlying metabolic syndrome.

Studies in animal models have helped clarify how different types of propolis influence key features of metabolic syndrome. In obese mice, daily intraperitoneal injections of artepillin C from Brazilian green propolis, given at 10 to 20 mg per kilogram of body weight for five weeks, lowered fasting glucose, improved insulin sensitivity, and reduced circulating lipids by interfering with the CREB/CRTC2 signaling complex [201]. A structurally optimized derivative, referred to as A57, produced even stronger metabolic improvements [201]. Additional work in C57BL/6 mice fed a high-fat diet showed that nine weeks of Chinese propolis supplementation at 150 to 300 mg per kilogram moderated body weight gain, reduced hepatic steatosis, and normalized gut microbial composition through changes in the expression of genes such as PGC1, SREBP1/2, and PPARα/γ [147]. In another model, Taiwanese green propolis provided at 65 to 150 mg per kilogram for twelve weeks enhanced glucose tolerance, improved insulin sensitivity, and increased microbial diversity [124]. Taken together, these studies suggest that the metabolic impact of propolis depends not only on dosage and treatment length but also on differences in the flavonoid composition of regional propolis varieties.

A systematic review and meta-analysis showed that taking between 250 and 900 mg of propolis per day for periods of six to twelve weeks lowered total cholesterol, LDL cholesterol, and triglyceride levels in people with metabolic syndrome or related metabolic disturbances, suggesting that short-term supplementation may be associated with improvements in lipid profiles [202]. In a randomized controlled trial conducted in Iran, participants who received 900 mg of propolis daily—divided into two doses of 450 mg before lunch and dinner—for twelve weeks experienced reductions in fasting glucose, triglycerides, body weight, and waist circumference. The benefits were more pronounced among individuals who also adhered to the MIND diet [203]. Another double-blind, placebo-controlled study using a daily dose of 500 mg over the same duration found decreases in waist circumference and improvements in quality of life measures such as physical function, but no significant effects on blood lipids or glucose levels [204]. While some trials reported broad metabolic and anthropometric improvements, others observed more limited changes. Such differences are likely influenced by variations in baseline metabolic status, population characteristics, and the length of the intervention.

Collectively, available studies suggest that propolis supplementation may be associated with improvements in selected metabolic syndrome-related parameters, most notably triglycerides and waist circumference, although findings are not uniform across trials. Evidence from animal models supports biological plausibility through reported effects on hepatic lipid handling, insulin sensitivity, inflammatory signaling, and gut microbiota composition. However, clinical studies remain limited in number and vary substantially in baseline metabolic status, dietary patterns, and co-interventions, which may partly explain inconsistent outcomes. Furthermore, differences in propolis type and standardization complicate the generalization of results. Therefore, while propolis may represent a promising adjunct in metabolic syndrome, further standardized and adequately powered trials aligned with established diagnostic criteria are necessary to clarify its clinical relevance.

### 5.4. Non-Alcoholic Fatty Liver Disease

NAFLD, recently termed MASLD, is strongly associated with central obesity, insulin resistance or type 2 diabetes, hypertension, and dyslipidemia. Its pathogenesis is largely driven by oxidative stress and chronic inflammation [205]. Propolis is increasingly being investigated for its potential to support liver health through antioxidant, anti-inflammatory, and metabolic regulatory actions. In recent years, both experimental and clinical studies have examined its effects on hepatic lipid accumulation, inflammation, and oxidative stress in NAFLD [206,207,208,209,210].

At the cellular level, in vitro studies provide additional insight into the hepatoprotective mechanisms of propolis. Kaempferol reduced intracellular triglycerides, total cholesterol, TNF-α, and IL-6 in oleic acid-stimulated HepG2 cells. In high-fat diet-fed rats, administration of 50 or 200 mg/kg/day for 16 weeks improved hepatic lipid profiles, enhanced antioxidant defenses (T-SOD and GSH), and reduced inflammatory cytokine expression, demonstrating dose-dependent effects [208]. Another study found that pretreating HepG2 and L02 hepatocytes with 25–100 μg/mL of Chinese propolis for 24 h protected against palmitic acid-induced oxidative stress and apoptosis by enhancing antioxidant enzymes (SOD, Nrf2, HO-1), restoring ATP levels, and suppressing TNF-α and IL-8 [207]. Together, these cellular, animal, and clinical findings provide a coherent mechanistic framework suggesting a potential role of propolis in NAFLD-related metabolic pathways.

These clinical observations are supported by mechanistic evidence from animal studies. In NAFLD-induced rats, oral administration of 100–200 mg/kg of propolis for two weeks improved serum lipid profiles, lowered ALT and ALP, suppressed pro-inflammatory cytokines such as IL-6 and TNF-α, and enhanced antioxidant capacity, as shown by decreased MDA and increased SH group levels. Histological analyses confirmed attenuation of liver injury [211]. Similar outcomes were observed in NASH mice fed a methionine–choline-deficient diet, where daily supplementation with 100 or 300 mg/kg of Brazilian propolis for eight weeks reduced ALT levels, improved liver histology, and suppressed ER stress-related gene expression [212]. These findings demonstrate that propolis and its bioactive components can influence multiple pathways relevant to NAFLD pathogenesis.

Although the number of clinical trials is limited, early findings provide preliminary evidence suggesting a potential supportive role of propolis in NAFLD. In a randomized, double-blind, placebo-controlled trial, daily supplementation with 1500 mg of Iranian propolis for eight weeks, combined with a calorie-restricted diet, prevented a decline in serum glutathione peroxidase but did not significantly affect other oxidative stress markers, body composition, or dietary antioxidant intake [209]. Another trial reported that daily supplementation with 450 mg of propolis for eight weeks significantly reduced ALT levels and improved hepatic steatosis, suggesting hepatoprotective effects through antioxidant and anti-inflammatory mechanisms [213]. Overall, these findings, though preliminary, indicate that propolis may offer supportive benefits in NAFLD treatment. Differences in sample size, intervention duration, and baseline metabolic status should be considered when interpreting these results.

In NAFLD/MASLD, early clinical trials suggest that propolis supplementation may be associated with improvements in liver enzymes and selected oxidative stress markers, while preclinical studies provide mechanistic support through modulation of inflammatory signaling, oxidative pathways, and ER stress responses. However, the clinical evidence remains preliminary, as trials are few, typically short in duration, and largely rely on biochemical outcomes rather than imaging- or histology-based endpoints. In addition, dietary restriction is frequently implemented concurrently, making it difficult to determine the independent contribution of propolis. Differences in baseline disease severity and propolis standardization further limit comparability across studies. Thus, although the current literature supports biological plausibility, more rigorous randomized trials with standardized preparations and clinically meaningful liver outcomes are required before definitive conclusions can be made.

## 6. Conclusions

The current literature highlights propolis as a natural bioactive substance with multifaceted biological activity and a potential supportive role in obesity and related metabolic disorders. Evidence from in vivo and in vitro studies suggests that its effects may be mediated through several key pathways, including reductions in oxidative stress and chronic inflammation, regulation of adipogenesis and lipid metabolism, and modulation of the gut microbiota. These mechanisms may contribute to improvements in markers of glycaemic control, lipid metabolism, inflammatory mediators, and liver-related outcomes in some settings. Mechanistic support is largely derived from in vitro and animal models, whereas human RCTs have mainly reported changes in surrogate metabolic biomarkers rather than consistent reductions in body weight or fat mass. However, outcomes related to body weight, body mass index, and overall fat mass have been variable across studies. Therefore, current evidence should be interpreted primarily as indicative of metabolic biomarker modulation rather than confirmed anti-obesity efficacy. Interpretation is further constrained by substantial heterogeneity in propolis origin, extraction method, chemical standardization, dosing regimens, and trial populations, underscoring the need for more rigorously designed clinical trials in obesity. Future obesity-focused RCTs should prioritize standardized preparations with defined polyphenol profiles, prespecified primary endpoints, adequate dietary control, and longer follow-up to clarify dose–response relationships and safety. The limited consistency of anthropometric outcomes underscores the need for adequately powered, long-term randomized controlled trials with standardized propolis preparations. As a scoping review, the primary objective was to map and synthesize the breadth of available mechanistic and clinical evidence rather than to provide a quantitative effect estimate.

However, the clinical evidence remains preliminary and heterogeneous, and several barriers still limit the translation of propolis into clinical practice. In particular, propolis preparations differ widely across studies with respect to botanical origin, geographic region, bee species, and extraction technique, resulting in substantial variability in chemical composition and bioactive content. As a consequence, comparability between trials is limited and dose–response interpretation remains challenging. Evidence for dose–response relationships remains limited in human obesity-focused trials, as most RCTs tested a single dose using heterogeneous preparations; however, preclinical studies and dose–response meta-analytic evidence suggest that certain metabolic endpoints (e.g., liver enzymes and glycaemic outcomes) may improve across a broad intake range. Moreover, most available clinical studies have been small and short in duration, frequently relying on surrogate metabolic biomarkers rather than robust adiposity endpoints. Collectively, these issues restrict the strength of inference and underscore the need for standardized, chemically characterized preparations in future clinical research.

Future clinical research should focus on standardized, chemically characterized propolis extracts. Larger and longer trials with clear metabolic endpoints are needed to confirm efficacy and safety. In particular, dose–response designs, robust adiposity measurements (e.g., visceral fat and body composition), and adequate dietary control will be essential to reduce heterogeneity and improve interpretability. Integrating phytochemical, clinical, and microbiota-focused approaches will help clarify mechanisms. Overall, propolis may have potential as a complementary strategy supporting metabolic health in obesity and related metabolic disorders, but well-designed and harmonized studies are essential to translate this promise into clinical practice, and current findings do not yet justify firm clinical recommendations.

Nevertheless, these findings should be interpreted in light of several limitations. As this work was conducted as a scoping review aiming to map the breadth of available evidence, it was not designed to provide quantitative effect estimates; therefore, a formal risk-of-bias assessment and meta-analysis were not performed. Furthermore, the included studies were highly heterogeneous in terms of design (in vitro, animal, and human studies), outcome measures, and reporting quality, which limits direct comparability and precludes strong causal inferences. Moreover, in a proportion of the included studies, key methodological details—such as extract characterization, polyphenol content, and intervention adherence—were insufficiently reported, which may have contributed to inconsistent findings across settings. Importantly, the overall safety profile of propolis cannot be generalized across preparations. Although most short-term clinical trials have reported good tolerability, adverse effects may occur, particularly in individuals with bee product allergies or atopic predisposition. Moreover, extraction solvent and formulation may influence both bioactive composition and safety outcomes, and isolated reports of hepatotoxicity—most often linked to ethanol-based extracts or concentrated products—warrant caution. Therefore, the current evidence base remains insufficient to support firm conclusions regarding long-term safety in humans. Long-term randomized safety data specifically in obesity populations remain unavailable, and future trials should incorporate systematic adverse-event monitoring and standardized safety reporting. Finally, the available human evidence remains limited with respect to long-term follow-up and safety reporting; thus, potential adverse events, particularly allergic reactions and hypersensitivity responses, should be carefully considered when interpreting clinical relevance.

## Figures and Tables

**Table 1:** Major propolis types: geographical distribution, botanical sources, and characteristic marker compounds.

Propolis Type	Key Marker Compounds	Botanical Source (Genus)	Geographical Distribution
Poplar-type	Chrysin, Pinocembrin, Galangin, CAPE, Caffeic acid, *p*-Coumaric acid	*Populus* spp. [40]	Europe, North America, Asia
Baccharis-type (Green)	Artepillin C, Kaempferide, *p*-Coumaric acid, Drupanin	*Baccharis dracunculifolia* [41]	Brazil
Red Propolis	Formononetin, Vestitol, Neovestitol, Isoliquiritigenin	*Dalbergia ecastophyllum* [42]	Brazil
Mediterranean-type	Quercetin, Apigenin, Luteolin, Pinobanksin	*Populus*, *Pinus* spp., and wild herbs [43]	Turkey, Greece, Italy
Dalbergia-type	Liquiritigenin, Isoliquiritigenin, Prenylated benzophenones	*Dalbergia* spp. [39]	Cuba, Venezuela

**Table 2:** Clinical trials of propolis supplementation: study characteristics, chemical standardization, and adiposity-related outcomes.

Study	Population	Baseline BMI/Adiposity	Study Design	Propolis Preparation/Standardization	Propolis Dose/Duration	Dietary Control	Primary Endpoint(s)	Main Findings
[152]	53 healthy women	BMI: 23.7 ± 0.7 (PLA) vs. 24.0 ± 0.6 (PRO); DXA fat%: 32.4 ± 1.2 vs. 33.0 ± 1.0	Double-blind RCT	Brazilian green propolis extract (Propolis 300^®^); standardization: NR.	454 mg/day for 12 weeks	Habitual diet and physical activity maintained; dietary intake not quantified (NR).	DXA fat mass (sample size calculated for fat mass)	↓ DXA fat mass (−0.3 kg vs. placebo) ↓ body fat % (−0.8%) ↑ adiponectin ↓ d-ROMs ↑ SOD (trend)
[153]	60 diabetic women with dyslipidemia	NR (reported anthropometrics included weight and WHR)	Single-blind, 4 arms	Propolis capsule (500 mg/day); origin/chemical standardization: NR	500 mg/day for 8 weeks	3-day 24 h dietary recall used to monitor intake	Lipid profile and oxidative stress markers (TAC), adiponectin	↑ TAC, ↑ adiponectin; ↓ TC ↓ TG ↓ LDL, ↓ IL-6
[154]	36 newly diagnosed T2DM patients	BMI: 25–34.9 kg/m^2^; baseline BMI: NR	Double-blind, placebo-controlled RCT	Commercial propolis (NOW; purity > 95%); markers/polyphenols: NR.	2 × 300 mg/day for 12 weeks	NR (if not described as food record/recall)	Glycemic indices (FPG, HbA1c) and insulin sensitivity	↓ FPG vs. placebo (*p* = 0.004) ↓ HbA1c vs. placebo (*p* = 0.049) ↓ 2 h PG (within-group, *p* < 0.05) ↑ insulin sensitivity (Matsuda index)
[155]	44 obese NAFLD patients	No significant between-group change in BMI or weight	Double-blind RCT	Propolis supplement; chemical standardization: NR	500 mg/day for 8 weeks	Calorie-restricted diet (−500 kcal/day) + 3-day food record	Glycemic indices (insulin, HOMA-IR, FPG), NAFLD score, QUICKI	↓ FBS (*p* = 0.037) ↓ Insulin (*p* = 0.040) ↓ HOMA-IR (*p* = 0.007) ↑ QUICKI (*p* = 0.015) ↓ NAFLD fibrosis score (*p* = 0.013); NNT ≈ 3
[156]	66 T2DM patients	BMI: NR (unless baseline table provides)	RCT	Propolis supplement; origin/standardization: NR	900 mg/day for 12 weeks	NR	Glycemic control (FPG, HbA1c)	↓ FBG (−17.8 mg/dL vs. +6.5 mg/dL placebo, *p* = 0.01) ↓ HbA1c (*p* < 0.05) ↓ TC (*p* = 0.01) ↓ LDL (*p* < 0.05)
[157]	50 T2DM patients with chronic periodontitis	BMI: NR (unless baseline table provides)	RCT	BioPropolis^®^ (IBE Pharma, Cairo, Egypt); chemical standardization: NR	400 mg/day for 6 months	NR	HbA1c (primary); periodontal healing parameters	↓ HbA1c (−0.96%, 6 months, *p* < 0.01) ↓ FPG (*p* < 0.05)
[158]	65 patients with T2DM	BMI measured (baseline BMI available in paper; if not in table: NR)	RCT	Brazilian green propolis ethanol extract; chemical standardization: NR	900 mg/day for 18 weeks	5-day 24 h dietary recall	Inflammation/oxidative stress (TNF-α, GSH, polyphenols), glycemic indices (HbA1c)	No significant between-group difference in FBG or HbA1c ↑ GSH (within-group, *p* < 0.05) ↑ total polyphenols (within-group, *p* < 0.05) ↓ carbonyls (within-group, *p* < 0.05) ↓ TNF-α (within-group, *p* < 0.05)

Arrows indicate the direction of change relative to baseline or the control/placebo group (↑ increase, ↓ decrease). Where effect sizes were not reported, outcomes are summarized by direction of change (↑/↓). Abbreviations: RCT, randomized controlled trial; T2DM, type 2 diabetes mellitus; NAFLD, non-alcoholic fatty liver disease; FPG, fasting plasma glucose; HbA1c, glycated hemoglobin; HOMA-IR, homeostatic model assessment of insulin resistance; QUICKI, quantitative insulin sensitivity check index. Oxidative stress/inflammatory markers: SOD, superoxide dismutase; TAC, total antioxidant capacity; GSH, reduced glutathione; d-ROMs, derivatives of reactive oxygen metabolites; IL-6, interleukin-6; TNF-α, tumor necrosis factor-α. NR indicates not reported in the original publication. Several trials did not provide baseline adiposity indices or chemical standardization parameters (e.g., total polyphenol/flavonoid content or marker compounds), which may contribute to heterogeneity across outcomes.

**Table 3:** Experimental animal studies of whole propolis preparations in obesity-related metabolic outcomes.

Study	Animal Model	Intervention	Dose/Route/Duration	Experimental Setup	Metabolic Outcomes	Mechanisms/Key Findings
[159]	Mice (long-term intake)	Chinese propolis (dietary)	200 mg/kg/day/drinking water/16 weeks	Long-term feeding model	↓ Adiposity, altered liver lipid composition	Phospholipid remodeling; altered glycerolipid metabolism.
[160]	HFD-fed mice	Ethanolic extract of propolis (EEP)	50 mg/kg/day/oral gavage/ 30 days	Hyperlipidemia model	↓ TG, ↓ LDL, ↑ HDL, ↓ Atherogenic index	Improved lipid profile; ↑ antioxidant markers.
[161]	STZ-induced diabetic rats	Propolis vs. nano-propolis	100 mg/kg/day/oral administration/60 days	Diabetic + standard vs. nano-formulations	↑ GSH, ↓ MDA, ↑ Testosterone, ↑ Sperm quality, ↑ CYP11A1, ↑ HSD-3β	Nano-formulation vs. crude: stronger antioxidant and steroidogenesis-related outcomes.
[162]	STZ-diabetic rats	Egyptian propolis	300 mg/kg/day/oral gavage/28 days	Diabetic nephropathy model	↓ Renal markers, ↓ oxidative stress, improved histology	Renoprotection; ↓ oxidative stress; improved histology.
[163]	Diabetic rats	Malaysian propolis	300 mg/kg/day/oral administration/4 weeks	STZ-induced diabetes	↓ ALT/AST, ↓ MDA, ↓ IL-6, ↓ TNF-α	Hepatoprotection; antioxidant and anti-inflammatory activity.
[164]	Diabetic pregnant rats	Malaysian propolis	300 mg/kg/day/oral administration/4 weeks	STZ-induced gestational diabetes	↓ FPG, ↑ conception rate, ↓ oxidative stress in placenta	Improved pregnancy outcomes; ↓ placental oxidative stress markers.
[165]	STZ-induced diabetic rats	Chinese and Brazilian propolis	100 mg/kg/day/oral administration/twice daily for 8 weeks	STZ-induced diabetes model	↓ Body weight loss ↓ Fasting blood glucose, ↓ HbA1c, ↓ Total cholesterol, ↓ triglycerides	Improved glycemic and lipid parameters; enhanced antioxidant activity and metabolic regulation.

Arrows indicate the direction of change compared with control groups (↑ increase, ↓ decrease). Mechanistic terms reflect pathways and biomarkers reported in the original studies. Abbreviations: HFD, high-fat diet; STZ, streptozotocin; FPG, fasting plasma glucose; ALT, alanine aminotransferase; AST, aspartate aminotransferase; TG, triglycerides; LDL, low-density lipoprotein cholesterol; HDL, high-density lipoprotein cholesterol; GSH, reduced glutathione; MDA, malondialdehyde; IL-6, interleukin-6; TNF-α, tumor necrosis factor-α; HbA1c, glycated hemoglobin.

**Table 4:** Experimental animal studies of isolated propolis-derived compounds in obesity-related metabolic outcomes.

Study	Animal Model	Intervention	Dose & Duration	Experimental Setup	Metabolic Outcomes	Mechanisms/Key Findings
[166]	Obese rats (HFD-induced)	Chrysin	100 mg/kg/day/oral gavage/8 weeks	Control, HFD-obese, chrysin, swimming, combo	↓ Weight, ↓ Insulin, ↓ TNF-α, ↑ PPAR-γ, ↓ miR-27a, ↓ MDA	↓ weight gain; ↓ inflammatory markers; strongest effects with combined swimming.
[167]	HFD-induced obese mice	Pinocembrin (propolis flavonoid)	20 mg/kg/day/oral gavage/10 weeks	HFD ± pinocembrin	↓ Weight, ↑ Insulin sensitivity, ↓ adipose inflammation	Improved metabolic outcomes; GPR120/ERK1/2 pathway modulation.
[168]	HFD-induced obese mice	Chrysin (a flavonoid from propolis)	60–200 mg/kg/oral gavage/4–10 weeks	Diet-induced obesity & inflammation	↓ Glucose, ↓ insulin, ↓ inflammation	Improved insulin sensitivity; IKK/TBK1 pathway modulation.
[169]	HFD-fed rats	Chrysin (flavonoid)	100 mg/kg/day, 30 days (route NR)	Diet-induced dyslipidemia	↓ ALT/AST/CK-MB; ↓ TC, TG, LDL, VLDL; ↓ ROS & inflammatory genes; ↑ Nrf2 & eNOS	Antioxidant and anti-inflammatory effects; vascular protection; possible microbiota involvement.
[170]	C57BL/6J mice	Artepillin C (Brazilian propolis component)	10 mg/kg/day/oral administration/28 days	Cold exposure-induced thermogenesis model	↑ iWAT thermogenesis, ↑ core temperature	↑ thermogenesis; beige adipocyte markers; creatine-metabolism pathways implicated.
[171]	Diabetic mice (HFD/STZ)	Tectochrysin (propolis flavonoid)	10–30 mg/kg/day/oral gavage/4 weeks	Type 2 diabetes model (diet + STZ)	↓ Glucose, improved lipid profile	Improved insulin sensitivity; AMPK activation.
[172]	Ethanol-fed mice	Galangin (propolis flavonoid)	50 mg/kg/day/oral gavage/14 days	Alcohol-induced gut barrier damage	↑ Gut barrier, ↓ IL-1β, ↑ SCFAs	↓ NLRP3 inflammasome activation; improved gut barrier markers.
[173]	C57BL/6 mice	CAPE (from propolis)	75 mg/kg/day/oral gavage/8 weeks	HFD-induced NAFLD	↓ Bile acids, ↑ FXR, ↑ GLP-1, improved liver histology	Microbiota–FXR axis modulation; ceramide-related pathways implicated.

Arrows indicate the direction of change compared with control groups (↑ increase, ↓ decrease). Mechanistic terms reflect pathways and biomarkers reported in the original studies. Abbreviations: HFD, high-fat diet; STZ, streptozotocin; iWAT, inguinal white adipose tissue; ALT, alanine aminotransferase; AST, aspartate aminotransferase; TC, total cholesterol; TG, triglycerides; LDL, low-density lipoprotein cholesterol; CK-MB, creatine kinase-MB; MDA, malondialdehyde; TNF-α, tumor necrosis factor-α; SCFAs, short-chain fatty acids; FXR, farnesoid X receptor; GLP-1, glucagon-like peptide-1; AMPK, AMP-activated protein kinase; NLRP3, NOD-like receptor pyrin domain-containing protein 3; eNOS, endothelial nitric oxide synthase.

## Data Availability

No new data were created or analyzed in this study.

## Supplementary Materials

The following supporting information can be downloaded at: https://www.mdpi.com/article/10.3390/nu18050826/s1, Table S1: Detailed Electronic Search Strategy; Supplementary Material S2: Completed PRISMA-ScR Checklist.

## References

[1] World Health Organization (WHO) One in Eight People Are Now Living with Obesity. https://www.who.int/news/item/01-03-2024-one-in-eight-people-are-now-living-with-obesity.

[2] Hay S.I. (2025). Global, regional, and national prevalence of adult overweight and obesity, 1990–2021, with forecasts to 2050: A forecasting study for the global burden of disease study 2021. Lancet.

[3] Kloock S., Ziegler C.G., Dischinger U. (2023). Obesity and its comorbidities, current treatment options and future perspectives: Challenging bariatric surgery?. Pharmacol. Ther..

[4] Aruwa C.E., Sabiu S. (2024). Adipose tissue inflammation linked to obesity: A review of current understanding, therapies and relevance of phyto-therapeutics. Heliyon.

[5] Ahmad B., Friar E.P., Vohra M.S., Garrett M.D., Serpell C.J., Fong I.L., Wong E.H. (2020). Mechanisms of action for the anti-obesogenic activities of phytochemicals. Phytochemistry.

[6] Galassetti P. (2012). Inflammation and Oxidative Stress in Obesity, Metabolic Syndrome, and Diabetes. Exp. Diabetes Res..

[7] McArdle M.A., Finucane O.M., Connaughton R.M., McMorrow A.M., Roche H.M. (2013). Mechanisms of obesity-induced inflammation and insulin resistance: Insights into the emerging role of nutritional strategies. Front. Endocrinol..

[8] Apovian C.M. (2016). Obesity: Definition, Comorbidities, Causes, and Burden. Am. J. Manag. Care.

[9] Kumar M., Kaushik D., Kaur J., Proestos C., Oz F., Oz E., Gupta P., Kundu P., Kaur A., Anisha A. (2022). A critical review on obesity: Herbal approach, bioactive compounds, and their mechanism. Appl. Sci..

[10] Ramírez-Moreno E., Arias-Rico J., Jiménez-Sánchez R.C., Estrada-Luna D., Jiménez-Osorio A.S., Zafra-Rojas Q.Y., Ariza-Ortega J.A., Flores-Chávez O.R., Morales-Castillejos L., Sandoval-Gallegos E.M. (2022). Role of Bioactive compounds in obesity: Metabolic mechanism focused on inflammation. Foods.

[11] Suran J., Cepanec I., Masek T., Radic B., Radic S., Gajger I.T., Vlainic J. (2021). Propolis extract and its bioactive compounds-from traditional to modern extraction technologies. Molecules.

[12] Zhu L., Zhang J., Yang H., Li G., Li H., Deng Z., Zhang B. (2023). Propolis polyphenols: A review on the composition and anti-obesity mechanism of different types of propolis polyphenols. Front. Nutr..

[13] Zullkiflee N., Taha H., Usman A. (2022). Propolis: Its role and efficacy in human health and diseases. Molecules.

[14] Zulhendri F., Lesmana R., Tandean S., Christoper A., Chandrasekaran K., Irsyam I., Suwantika A.A., Abdulah R., Wathoni N. (2022). Recent update on the anti-inflammatory activities of propolis. Molecules.

[15] Almuhayawi M.S. (2020). Propolis as a novel antibacterial agent. Saudi J. Biol. Sci..

[16] Touzani S., Embaslat W., Imtara H., Kmail A., Kadan S., Zaid H., ElArabi I., Badiaa L., Saad B. (2019). Evaluation of the potential use of propolis as a multitarget therapeutic product: Physicochemical properties, chemical composition, and immunomodulatory, antibacterial and anticancer properties. Biomed Res. Int..

[17] Goncalves V.C., Fonseca V.S.D., Faria D.D., Izidoro M.A., Berretta A.A., Almeida A.C.G.D., Fonseca F.L.A., Scorza F.A., Scorza C.A. (2022). Propolis induces cardiac metabolism changes in 6-hydroxydopamine animal model: A dietary intervention as a potential cardioprotective approach in parkinson’s disease. Front. Pharmacol..

[18] El-Kersh D.M., Abou El-Ezz R.F., Ramadan E., El-kased R.F. (2024). *In Vitro* and *in vivo* burn healing study of standardized propolis: Unveiling its antibacterial, antioxidant and anti-inflammatory actions in relation to its phytochemical profiling. PLoS ONE.

[19] Barber T.M., Kabisch S., Randeva H.S., Pfeiffer A.F.H., Weickert M.O. (2022). Implications of resveratrol in obesity and insulin resistance: A state-of-the-art review. Nutrients.

[20] Casanova E., Salvadó J., Crescenti A., Gibert-Ramos A. (2019). Epigallocatechin gallate modulates muscle homeostasis in type 2 diabetes and obesity by targeting energetic and redox pathways: A narrative review. Int. J. Mol. Sci..

[21] Farhat G., Drummond S., Al-Dujaili E.A.S. (2017). Polyphenols and Their role in obesity management: A systematic review of randomized clinical trials. Phytother. Res..

[22] Mehta J., Rayalam S., Wang X.Y. (2018). Cytoprotective Effects of natural compounds against oxidative stress. Antioxidants.

[23] Yi R.H., Liu Y., Zhang X., Sun X.C., Wang N.N., Zhang C., Deng H.Y., Yao X.F., Wang S.P., Yang G. (2024). Unraveling quercetin’s potential: A comprehensive review of its properties and mechanisms of action, in diabetes and obesity complications. Phytother. Res..

[24] Tricco A.C., Lillie E., Zarin W., O’Brien K.K., Colquhoun H., Levac D., Moher D., Peters M.D.J., Horsley T., Weeks L. (2018). PRISMA extension for scoping reviews (PRISMA-ScR): Checklist and explanation. Ann. Intern. Med..

[25] Arksey H., O’Malley L. (2005). Scoping studies: Towards a methodological framework. Int. J. Soc. Res. Methodol..

[26] Levac D., Colquhoun H., O’Brien K.K. (2010). Scoping studies: Advancing the methodology. Implement. Sci..

[27] Zabaiou N., Fouache A., Trousson A., Baron S., Zellagui A., Lahouel M., Lobaccaro J.M.A. (2017). Biological properties of propolis extracts: Something new from an ancient product. Chem. Phys. Lipids.

[28] Przybylek I., Karpinski T.M. (2019). Antibacterial properties of propolis. Molecules.

[29] Kuropatnicki A.K., Szliszka E., Krol W. (2013). Historical aspects of propolis research in modern times. Evid. Based Complement. Alternat Med..

[30] Anjum S.I., Ullah A., Khan K.A., Attaullah M., Khan H., Ali H., Bashir M.A., Tahir M., Ansari M.J., Ghramh H.A. (2019). Composition and functional properties of propolis (bee glue): A review. Saudi J. Biol. Sci..

[31] Forma E., Brys M. (2021). Anticancer activity of propolis and its compounds. Nutrients.

[32] Alday E., Valencia D., Garibay-Escobar A., Dominguez-Esquivel Z., Piccinelli A.L., Rastrelli L., Monribot-Villanueva J., Guerrero-Analco J.A., Robles-Zepeda R.E., Hernandez J. (2019). Plant origin authentication of Sonoran Desert propolis: An antiproliferative propolis from a semi-arid region. Naturwissenschaften.

[33] Hossain R., Quispe C., Khan R.A., Saikat A.S.M., Ray P., Ongalbek D., Yeskaliyeva B., Jain D., Smeriglio A., Trombetta D. (2022). Propolis: An update on its chemistry and pharmacological applications. Chin. Med..

[34] Salleh S.N.A.S., Wan Lim W.M., Hanapiah N.A.M. (2022). A comprehensive review on chemical compounds, biological actions and potential health benefits of stingless bee propolis. Sains Malaysiana.

[35] Salatino A., Teixeira E.W., Negri G., Message D. (2005). Origin and chemical variation of Brazilian propolis. Evid. Based Complement. Alternat Med..

[36] Ristivojevic P., Trifkovic J., Andric F., Milojkovic-Opsenica D. (2015). Poplar-type propolis: Chemical composition, botanical origin and biological activity. Nat. Prod. Commun..

[37] Bastos E.M., Santana R.A., Calaca-Costa A.G., Thiago P.S. (2011). Interaction between Apis mellifera L. and Baccharis dracunculifolia DC, that favours green propolis production in Minas Gerais. Braz. J. Biol..

[38] Rajan M.B., Tavares D., de Oliveira C.S., de Oliveira D.G., Narain N. (2018). Optimization of solvent extraction and HPLC-DAD method parameters for determination of phenolic compounds in various Brazilian propolis. J. Food Sci. Technol..

[39] Ccana-Ccapatinta G.V., Mejía J.A.A., Tanimoto M.H., Groppo M., Carvalho J., Bastos J.K. (2020). *Dalbergia ecastaphyllum* (L.) Taub. and *Symphonia globulifera* L.f.: The botanical sources of isoflavonoids and benzophenones in Brazilian red propolis. Molecules.

[40] Dezmirean D.S., Paşca C., Moise A.R., Bobiş O. (2020). Plant sources responsible for the chemical composition and main bioactive properties of poplar-type propolis. Plants.

[41] Park Y.K., Alencar S.M., Aguiar C.L. (2002). Botanical origin and chemical composition of Brazilian propolis. J. Agric. Food Chem..

[42] Silva B.B., Rosalen P.L., Cury J.A., Ikegaki M., Souza V.C., Esteves A., Alencar S.M. (2008). Chemical composition and botanical origin of red propolis, a new type of Brazilian propolis. Evid.-Based Complement. Altern..

[43] Stavropoulou M.I., Stathopoulou K., Cheilari A., Benaki D., Gardikis K., Chinou I., Aligiannis N. (2021). NMR metabolic profiling of Greek propolis samples: Comparative evaluation of their phytochemical compositions and investigation of their anti-ageing and antioxidant properties. J. Pharmaceut. Biomed..

[44] Trusheva B., Popova M., Bankova V., Simova S., Marcucci M.C., Miorin P.L., da Rocha Pasin F., Tsvetkova I. (2006). Bioactive constituents of Brazilian red propolis. Evid. Based Complement. Altern. Med..

[45] Santos L.M., Fonseca M.S., Sokolonski A.R., Deegan K.R., Araujo R.P., Umsza-Guez M.A., Barbosa J.D., Portela R.D., Machado B.A. (2020). Propolis: Types, composition, biological activities, and veterinary product patent prospecting. J. Sci. Food Agric..

[46] Regueira M.S.N., Tintino S.R., da Silva A.R.P., Costa M.D.S., Boligon A.A., Matias E.F.F., de Queiroz Balbino V., Menezes I.R.A., Melo Coutinho H.D. (2017). Seasonal variation of Brazilian red propolis: Antibacterial activity, synergistic effect and phytochemical screening. Food Chem. Toxicol..

[47] Paulino N., Abreu S.R., Uto Y., Koyama D., Nagasawa H., Hori H., Dirsch V.M., Vollmar A.M., Scremin A., Bretz W.A. (2008). Anti-inflammatory effects of a bioavailable compound, Artepillin C, in Brazilian propolis. Eur. J. Pharmacol..

[48] Seibert J.B., Bautista-Silva J.P., Amparo T.R., Petit A., Pervier P., Dos Santos Almeida J.C., Azevedo M.C., Silveira B.M., Brandao G.C., de Souza G.H.B. (2019). Development of propolis nanoemulsion with antioxidant and antimicrobial activity for use as a potential natural preservative. Food Chem..

[49] Kucukler S., Benzer F., Yildirim S., Gur C., Kandemir F.M., Bengu A.S., Ayna A., Caglayan C., Dortbudak M.B. (2021). Protective effects of chrysin against oxidative stress and inflammation induced by lead acetate in rat kidneys: A biochemical and histopathological approach. Biol. Trace Elem. Res..

[50] Gebhard C., Stahli B.E., Largiader S., Holy E.W., Akhmedov A., Camici G.G., Luscher T.F., Tanner F.C. (2013). Caffeic acid phenethyl ester inhibits endothelial tissue factor expression. Biol. Pharm. Bull..

[51] Rivera L., Moron R., Sanchez M., Zarzuelo A., Galisteo M. (2008). Quercetin ameliorates metabolic syndrome and improves the inflammatory status in obese Zucker rats. Obesity.

[52] Kumar S., Alagawadi K.R. (2013). Anti-obesity effects of galangin, a pancreatic lipase inhibitor in cafeteria diet fed female rats. Pharm. Biol..

[53] Gomez-Caravaca A.M., Gomez-Romero M., Arraez-Roman D., Segura-Carretero A., Fernandez-Gutierrez A. (2006). Advances in the analysis of phenolic compounds in products derived from bees. J. Pharm. Biomed. Anal..

[54] Devequi-Nunes D., Machado B.A.S., Barreto G.A., Reboucas Silva J., da Silva D.F., da Rocha J.L.C., Brandao H.N., Borges V.M., Umsza-Guez M.A. (2018). Chemical characterization and biological activity of six different extracts of propolis through conventional methods and supercritical extraction. PLoS ONE.

[55] Burdock G.A. (1998). Review of the biological properties and toxicity of bee propolis (propolis). Food Chem. Toxicol..

[56] Brailo V., Boras V.V., Alajbeg I., Juras V. (2006). Delayed contact sensitivity on the lips and oral mucosa due to propolis-case report. Med. Oral Patol. Oral Cir. Bucal.

[57] Arvouet-Grand A., Lejeune B., Bastide P., Pourrat A., Legret P. (1993). Propolis extract. Part 6. Subacute toxicity and cutaneous primary irritation index. Drug Chem. Toxicol..

[58] Hollands I., Vázquez A., Gra B., Sotolongo M. (1994). Evaluation of the subchronic toxicity of Cuban propolis. J. Ethnopharmacol..

[59] Gritsenko V.I., Tikhonov O.I., Priakhin O.R. (1977). Study of the polysaccharide preparation, propolis. Farm. Zh..

[60] Christoper A., Herman H., Abdulah R., Zulhendri F., Lesmana R. (2025). Short Communication: The Effect of propolis extract treatment on the Lee index and brain-body weight ratio in diet-induced obesity rats. Biochem. Biophys. Rep..

[61] Tian S., Zhao H., Guo H., Feng W., Jiang C., Jiang Y. (2023). Propolis ethanolic extract attenuates D-gal-induced C2C12 cell injury by modulating Nrf2/HO-1 and p38/p53 signaling pathways. Int. J. Mol. Sci..

[62] Chen Y.W., Chen Y.H., Yu Y.H. (2018). Taiwanese green propolis ethanol extract promotes adipocyte differentiation and alleviates TNF-α-mediated down-regulation of adiponectin expression. Evid. Based Complement. Altern. Med..

[63] Yadav R., Swetanshu, Singh P. (2024). The molecular mechanism of obesity: The science behind natural exercise yoga and healthy diets in the treatment of obesity. Curr. Probl. Cardiol..

[64] Nicze M., Dec A., Borowka M., Krzyzak D., Boldys A., Buldak L., Okopien B. (2024). Molecular mechanisms behind obesity and their potential exploitation in current and future therapy. Int. J. Mol. Sci..

[65] Wu Q., Li J., Hao S., Guo Y., Li Z., Liu Z., Xuan H. (2023). Caffeic acid phenethyl ester inhibits MDA-MB-231 cell proliferation in inflammatory microenvironment by suppressing glycolysis and lipid metabolism. Biomed. Pharmacother..

[66] Kitamura H., Saito N., Fujimoto J., Nakashima K.I., Fujikura D. (2018). Brazilian propolis ethanol extract and its component kaempferol induce myeloid-derived suppressor cells from macrophages of mice *in vivo* and *in vitro*. BMC Complement. Altern. Med..

[67] El Adaouia Taleb R., Djebli N., Chenini H., Sahin H., Kolayli S. (2020). *In vivo* and *in vitro* anti-diabetic activity of ethanolic propolis extract. J. Food Biochem..

[68] Iio A., Ohguchi K., Inoue H., Maruyama H., Araki Y., Nozawa Y., Ito M. (2010). Ethanolic extracts of Brazilian red propolis promote adipocyte differentiation through Pparγ activation. Phytomedicine.

[69] Bhatti J.S., Bhatti G.K., Reddy P.H. (2017). Mitochondrial dysfunction and oxidative stress in metabolic disorders—A step towards mitochondria based therapeutic strategies. Biochim. Biophys. Acta Mol. Basis Dis..

[70] Masschelin P.M., Cox A.R., Chernis N., Hartig S.M. (2019). The Impact of oxidative stress on adipose tissue energy balance. Front. Physiol..

[71] Manna P., Jain S.K. (2015). Obesity, oxidative stress, adipose tissue dysfunction, and the associated health risks: Causes and therapeutic strategies. Metab. Syndr. Relat. Disord..

[72] Houstis N., Rosen E.D., Lander E.S. (2006). Reactive oxygen species have a causal role in multiple forms of insulin resistance. Nature.

[73] Furukawa S., Fujita T., Shimabukuro M., Iwaki M., Yamada Y., Nakajima Y., Nakayama O., Makishima M., Matsuda M., Shimomura I. (2004). Increased oxidative stress in obesity and its impact on metabolic syndrome. J. Clin. Investig..

[74] Kitamura H. (2019). Effects of propolis extract and propolis-derived compounds on obesity and diabetes: Knowledge from cellular and animal models. Molecules.

[75] Tsai Y.C., Wang Y.H., Liou C.C., Lin Y.C., Huang H., Liu Y.C. (2012). Induction of oxidative DNA damage by flavonoids of propolis: Its mechanism and implication about antioxidant capacity. Chem. Res. Toxicol..

[76] Russo A., Longo R., Vanella A. (2002). Antioxidant activity of propolis: Role of caffeic acid phenethyl ester and galangin. Fitoterapia.

[77] Zheng Y.Z., Deng G., Liang Q., Chen D.F., Guo R., Lai R.C. (2017). Antioxidant activity of quercetin and its glucosides from propolis: A theoretical study. Sci. Rep..

[78] Bea F., Hudson F.N., Chait A., Kavanagh T.J., Rosenfeld M.E. (2003). Induction of glutathione synthesis in macrophages by oxidized low-density lipoproteins is mediated by consensus antioxidant response elements. Circ. Res..

[79] Zhang J., Cao X., Ping S., Wang K., Shi J., Zhang C., Zheng H., Hu F. (2015). Comparisons of ethanol extracts of chinese propolis (poplar type) and poplar gums based on the antioxidant activities and molecular mechanism. Evid. Based Complement. Altern. Med..

[80] Scorza C., Goncalves V., Finsterer J., Scorza F., Fonseca F. (2024). Exploring the prospective role of propolis in modifying aging hallmarks. Cells.

[81] Nadia B.H., Wided K., Kheira B., Hassiba R., Lamia B., Rhouati S., Alyane M., Zellagui A., Lahouel M. (2009). Disruption of mitochondrial membrane potential by ferulenol and restoration by propolis extract: Antiapoptotic role of propolis. Acta Biol. Hung..

[82] Balion Z., Ramanauskiene K., Jekabsone A., Majiene D. (2020). The role of mitochondria in brain cell protection from ischaemia by differently prepared propolis extracts. Antioxidants.

[83] Nascimento T.S., Silva I.S.M., Alves M., Gouveia B.B., Barbosa L.M.R., Macedo T.J.S., Santos J.M.S., Monte A.P.O., Matos M.H.T., Padilha F.F. (2019). Effect of red propolis extract isolated or encapsulated in nanoparticles on the *in vitro* culture of sheep preantral follicle: Impacts on antrum formation, mitochondrial activity and glutathione levels. Reprod. Domest. Anim..

[84] Bittencourt M.L.F., Ribeiro P.R., Franco R.L.P., Hilhorst H.W.M., de Castro R.D., Fernandez L.G. (2015). Metabolite profiling, antioxidant and antibacterial activities of Brazilian propolis: Use of correlation and multivariate analyses to identify potential bioactive compounds. Food Res. Int..

[85] Cardinault N., Tourniaire F., Astier J., Couturier C., Perrin E., Dalifard J., Seipelt E., Mounien L., Letullier C., Bonnet L. (2020). Poplar propolis ethanolic extract reduces body weight gain and glucose metabolism disruption in high-fat diet-fed mice. Mol. Nutr. Food Res..

[86] Cardinault N., Tourniaire F., Astier J., Couturier C., Bonnet L., Seipelt E., Karkeni E., Letullier C., Dlalah N., George S. (2021). Botanic origin of propolis extract powder drives contrasted impact on diabesity in high-fat-fed mice. Antioxidants.

[87] Dinkova-Kostova A.T., Abramov A.Y. (2015). The emerging role of Nrf2 in mitochondrial function. Free Radic. Biol. Med..

[88] Piantadosi C.A., Suliman H.B. (2012). Redox regulation of mitochondrial biogenesis. Free Radic. Biol. Med..

[89] Zhang Y., Deng Q., Hong H., Qian Z., Wan B., Xia M. (2024). Caffeic acid phenethyl ester inhibits neuro-inflammation and oxidative stress following spinal cord injury by mitigating mitochondrial dysfunction via the SIRT1/PGC1alpha/DRP1 signaling pathway. J. Transl. Med..

[90] Heshmatipour H., Vajdi M., Tabrizi F.P.F., Golpour-Hamedani S., Askari G. (2025). Effects of propolis supplementation on inflammation and oxidative stress markers: A GRADE-assessed systematic review and meta-analysis of clinical trials. J. Funct. Foods.

[91] Bahari H., Shahraki Jazinaki M., Aliakbarian M., Rashidmayvan M., Golafrouz H., Rahnama I., Khodashahi R., Malekahmadi M. (2025). Propolis supplementation on inflammatory and oxidative stress biomarkers in adults: A systematic review and meta-analysis of randomized controlled trials. Front. Nutr..

[92] Toreti V.C., Sato H.H., Pastore G.M., Park Y.K. (2013). Recent progress of propolis for its biological and chemical compositions and its botanical origin. Evid. Based Complement. Altern. Med..

[93] Silva-Carvalho R., Baltazar F., Almeida-Aguiar C. (2015). Propolis: A complex natural product with a plethora of biological activities that can be explored for drug development. Evid. Based Complement. Altern. Med..

[94] Wieczorek P.P., Hudz N., Yezerska O., Horcinova-Sedlackova V., Shanaida M., Korytniuk O., Jasicka-Misiak I. (2022). Chemical variability and pharmacological potential of propolis as a source for the development of new pharmaceutical products. Molecules.

[95] Cox A.J., West N.P., Cripps A.W. (2015). Obesity, inflammation, and the gut microbiota. Lancet Diabetes Endocrinol..

[96] Gkrinia E.M.M., Belancic A. (2025). The mechanisms of chronic inflammation in obesity and potential therapeutic strategies: A narrative review. Curr. Issues Mol. Biol..

[97] Ahima R.S., Lazar M.A. (2008). Adipokines and the peripheral and neural control of energy balance. Mol. Endocrinol..

[98] Park H.S., Park J.Y., Yu R. (2005). Relationship of obesity and visceral adiposity with serum concentrations of CRP, TNF-α and IL-6. Diabetes Res. Clin. Pr..

[99] de Rooij S.R., Nijpels G., Nilsson P.M., Nolan J.J., Gabriel R., Bobbioni-Harsch E., Mingrone G., Dekker J.M. (2009). Low-grade chronic inflammation in the relationship between insulin sensitivity and cardiovascular disease (RISC) population associations with insulin resistance and cardiometabolic risk profile. Diabetes Care.

[100] Barateiro A., Mahú I., Domingos A.I. (2017). Leptin resistance and the neuro-adipose connection. Front. Endocrinol..

[101] Gan L.X., Guo K.Y., Cremona M.L., McGraw T.E., Leibel R.L., Zhang Y.Y. (2012). TNF-α up-regulates protein level and cell surface expression of the leptin receptor by stimulating its export via a PKC-dependent mechanism. Endocrinology.

[102] Wang B., Wood I.S., Trayhurn P. (2008). Hypoxia induces leptin gene expression and secretion in human preadipocytes: Differential effects of hypoxia on adipokine expression by preadipocytes. J. Endocrinol..

[103] Zhang Y.Y., Chua S. (2018). Leptin function and regulation. Compr. Physiol..

[104] Choi S.S., Cha B.Y., Iida K., Lee Y.S., Yonezawa T., Teruya T., Nagai K., Woo J.T. (2011). Artepillin C, as a PPARgamma ligand, enhances adipocyte differentiation and glucose uptake in 3T3-L1 cells. Biochem. Pharmacol..

[105] Song Y.S., Park E.H., Hur G.M., Ryu Y.S., Lee Y.S., Lee J.Y., Kim Y.M., Jin C. (2002). Caffeic acid phenethyl ester inhibits nitric oxide synthase gene expression and enzyme activity. Cancer Lett..

[106] Hotamisligil G.S. (2017). Inflammation, metaflammation and immunometabolic disorders. Nature.

[107] Lee Y.S., Huh J.Y., Hwang I., Kim J.I., Kim J.B. (2011). Hypoxia-mediated chronic inflammation is necessary for long term but not short term HFD-induced insulin resistance. Diabetes.

[108] Dos Santos F.F., Morais-Urano R.P., Cunha W.R., de Almeida S.G., Cavallari P., Manuquian H.A., Pereira H.A., Furtado R., Santos M.F.C., Amdrade E.S.M.L. (2022). A review on the anti-inflammatory activities of Brazilian green, brown and red propolis. J. Food Biochem..

[109] Boufadi M.Y., Soubhye J., Van Antwerpen P. (2021). Anti-inflammatory, antioxidant effects, and bioaccessibility of Tigzirt propolis. J. Food Biochem..

[110] Hsieh C.Y., Li L.H., Rao Y.K., Ju T.C., Nai Y.S., Chen Y.W., Hua K.F. (2019). Mechanistic insight into the attenuation of gouty inflammation by Taiwanese green propolis via inhibition of the NLRP3 inflammasome. J. Cell Physiol..

[111] Jalali M., Ranjbar T., Mosallanezhad Z., Mahmoodi M., Moosavian S.P., Ferns G.A., Jalali R., Sohrabi Z. (2020). Effect of propolis intake on serum c-reactive protein (CRP) and tumor necrosis factor-alpha (TNF-α) levels in adults: A systematic review and meta-analysis of clinical trials. Complement. Ther. Med..

[112] Abbasi E., Bagherniya M., Soleimani D., Ghasemi-Tehrani H., Abbaspour M., Clark C.C.T., Askari G. (2023). The effects of propolis supplementation on high-sensitivity C-reactive protein, testosterone hormone, and metabolic profile in women with polycystic ovary syndrome: A randomized, triple-blinded, placebo-controlled clinical trial. Phytother. Res..

[113] Soleimani D., Rezaie M., Rajabzadeh F., Gholizadeh Navashenaq J., Abbaspour M., Miryan M., Razmpour F., Ranjbar G., Rezvani R., Jarahi L. (2021). Protective effects of propolis on hepatic steatosis and fibrosis among patients with nonalcoholic fatty liver disease (NAFLD) evaluated by real-time two-dimensional shear wave elastography: A randomized clinical trial. Phytother. Res..

[114] Moreno M.J., Martinez J.A. (2002). Adipose tissue: A storage and secretory organ. An. Sist. Sanit. Navar..

[115] Lefterova M.I., Lazar M.A. (2009). New developments in adipogenesis. Trends Endocrinol. Metab..

[116] Tang Q.Q., Lane M.D. (2012). Adipogenesis: From stem cell to adipocyte. Annu. Rev. Biochem..

[117] Pei H., Yao Y., Yang Y., Liao K., Wu J.R. (2011). Kruppel-like factor KLF9 regulates PPARgamma transactivation at the middle stage of adipogenesis. Cell Death Differ..

[118] Christodoulides C., Lagathu C., Sethi J.K., Vidal-Puig A. (2009). Adipogenesis and WNT signalling. Trends Endocrinol. Metab..

[119] Kim G., Lee J., Ha J., Kang I., Choe W. (2023). Endoplasmic reticulum stress and its impact on adipogenesis: Molecular mechanisms implicated. Nutrients.

[120] Cho H., Kim K., Kim N., Woo M., Kim H.Y. (2020). Effect of propolis phenolic compounds on free fatty acid receptor 4 activation. Food Sci. Biotechnol..

[121] Kong L., Zhang Y., Feng Z., Dong J., Zhang H. (2021). Phenolic compounds of propolis alleviate lipid metabolism disorder. Evid. Based Complement. Altern. Med..

[122] Ahn S., Kim J., An S., Pyo J.J., Jung D., Lee J., Hwang S.Y., Gong J., Shin I., Kim H.P. (2019). 2-Phenyl-8-(1-phenylallyl)-chromenone compounds have a pan-PPAR modulator pharmacophore. Bioorg. Med. Chem..

[123] Nakashima K., Murakami T., Tanabe H., Inoue M. (2014). Identification of a naturally occurring retinoid X receptor agonist from Brazilian green propolis. Biochim. Biophys. Acta.

[124] Chien Y.H., Yu Y.H., Chen Y.W. (2023). Taiwanese green propolis ameliorates metabolic syndrome via remodeling of white adipose tissue and modulation of gut microbiota in diet-induced obese mice. Biomed. Pharmacother..

[125] Megantara I., Karisa P., Pakpahan W.I., Sylviana N., Goenawan H. (2025). Molecular mechanisms of propolis in adipogenesis, lipid metabolism and white adipose tissue browning: A systematic review of preclinical studies. Adipocyte.

[126] Nakajima M., Arimatsu K., Minagawa T., Matsuda Y., Sato K., Takahashi N., Nakajima T., Yamazaki K. (2016). Brazilian propolis mitigates impaired glucose and lipid metabolism in experimental periodontitis in mice. BMC Complement. Altern. Med..

[127] Ichi I., Hori H., Takashima Y., Adachi N., Kataoka R., Okihara K., Hashimoto K., Kojo S. (2009). The beneficial effect of propolis on fat accumulation and lipid metabolism in rats fed a high-fat diet. J. Food Sci..

[128] Huang X., Wu X., Yan S., Lan T. (2018). Lipid-lowering effect of propolis in mice with Triton-WR1339-induced hyperlipidemia and its mechanism for regulating lipid metabolism. Nan Fang Yi Ke Da Xue Xue Bao.

[129] Shin S.H., Seo S.G., Min S., Yang H., Lee E., Son J.E., Kwon J.Y., Yue S., Chung M.Y., Kim K.H. (2014). Caffeic acid phenethyl ester, a major component of propolis, suppresses high fat diet-induced obesity through inhibiting adipogenesis at the mitotic clonal expansion stage. J. Agric. Food Chem..

[130] Khoshandam A., Hedayatian A.H., Mollazadeh A.R., Razavi B.M., Hosseinzadeh H. (2023). Propolis and its constituents against cardiovascular risk factors including obesity, hypertension, atherosclerosis, diabetes, and dyslipidemia: A comprehensive review. Iran J. Basic Med. Sci..

[131] Mujica V., Orrego R., Perez J., Romero P., Ovalle P., Zuniga-Hernandez J., Arredondo M., Leiva E. (2017). The role of propolis in oxidative stress and lipid metabolism: A randomized controlled trial. Evid. Based Complement. Altern. Med..

[132] Zakerkish M., Jenabi M., Zaeemzadeh N., Hemmati A.A., Neisi N. (2019). The Effect of Iranian Propolis on glucose metabolism, lipid profile, insulin resistance, renal function and inflammatory biomarkers in patients with type 2 diabetes mellitus: A randomized double-blind clinical trial. Sci. Rep..

[133] Maddahi M., Nattagh-Eshtivani E., Jokar M., Barati M., Tabesh H., Safarian M., Khosravi M. (2023). The effect of propolis supplementation on cardiovascular risk factors in women with rheumatoid arthritis: A double-blind, placebo, controlled randomized clinical trial. Phytother. Res..

[134] Borrego-Ruiz A., Borrego J.J. (2025). The gut microbiome in human obesity: A comprehensive review. Biomedicines.

[135] Cheng Z., Zhang L., Yang L., Chu H. (2022). The critical role of gut microbiota in obesity. Front. Endocrinol..

[136] Liu Z., Li Z., Guo Y., Li Y., Xuan H. (2025). The protective effects of propolis against lipopolysaccharide-induced acute liver injury by modulating serum metabolites and gut flora. Sci. Rep..

[137] Aabed K., Shafi Bhat R., Moubayed N., Al-Mutiri M., Al-Marshoud M., Al-Qahtani A., Ansary A. (2019). Ameliorative effect of probiotics (*Lactobacillus paracaseii* and Protexin(R)) and prebiotics (propolis and bee pollen) on clindamycin and propionic acid-induced oxidative stress and altered gut microbiota in a rodent model of autism. Cell. Mol. Biol..

[138] Ayad A.S., Benchaabane S., Daas T., Smagghe G., Loucif-Ayad W. (2025). Propolis stands out as a multifaceted natural product: Meta-analysis on its sources, bioactivities, applications, and future perspectives. Life.

[139] Deng Y., Liu D., Dissanayake I., Jaye K., Bhuyan D.J., Low M., Li C.G. (2025). Propolis as a functional food ingredient: Modulation of gut microbiota and implications for chronic disease management. Food Res. Int..

[140] Garzarella E.U., Navajas-Porras B., Perez-Burillo S., Ullah H., Esposito C., Santarcangelo C., Hinojosa-Nogueira D., Pastoriza S., Zaccaria V., Xiao J. (2022). Evaluating the effects of a standardized polyphenol mixture extracted from poplar-type propolis on healthy and diseased human gut microbiota. Biomed. Pharmacother..

[141] Sun Y., Huang W., Shang Y., Sharaf El-Din M.G., Hang H., Wang P., Zhang C., Huang Y., Wang K. (2025). propolis modulates the gut microbiota-gut hormone-liver AMPK axis to ameliorate high-fat diet-induced metabolic disorders in rats. Nutrients.

[142] Wang K., Jin X., You M., Tian W., Le Leu R.K., Topping D.L., Conlon M.A., Wu L., Hu F. (2017). Dietary Propolis ameliorates dextran sulfate sodium-induced colitis and modulates the gut microbiota in rats fed a western diet. Nutrients.

[143] Guan R., Ma N., Liu G., Wu Q., Su S., Wang J., Geng Y. (2023). Ethanol extract of propolis regulates type 2 diabetes in mice via metabolism and gut microbiota. J. Ethnopharmacol..

[144] Xue M., Liu Y., Xu H., Zhou Z., Ma Y., Sun T., Liu M., Zhang H., Liang H. (2019). Propolis modulates the gut microbiota and improves the intestinal mucosal barrier function in diabetic rats. Biomed. Pharmacother..

[145] Byrne C.S., Chambers E.S., Morrison D.J., Frost G. (2015). The role of short chain fatty acids in appetite regulation and energy homeostasis. Int. J. Obes..

[146] Okamura T., Hamaguchi M., Bamba R., Nakajima H., Yoshimura Y., Kimura T., Hashimoto Y., Majima S., Senmaru T., Ushigome E. (2022). Brazilian green propolis improves gut microbiota dysbiosis and protects against sarcopenic obesity. J. Cachexia Sarcopenia Muscle.

[147] Zheng Y., Wu Y., Tao L., Chen X., Jones T.J., Wang K., Hu F. (2020). Chinese Propolis prevents obesity and metabolism syndromes induced by a high fat diet and accompanied by an altered gut microbiota structure in mice. Nutrients.

[148] Huang S., Yang X., Ma J., Li C., Wang Y., Wu Z. (2025). Ethanol extract of propolis relieves exercise-induced fatigue via modulating the metabolites and gut microbiota in mice. Front. Nutr..

[149] Kabali S., Unlu Sogut M., Oner N., Kara A. (2025). Protective effects of propolis supplementation on aflatoxin b1-induced oxidative stress, antioxidant status, intestinal barrier damage, and gut microbiota in rats. Mol. Nutr. Food Res..

[150] Fonseca L., Ribeiro M., Schultz J., Borges N.A., Cardozo L., Leal V.O., Ribeiro-Alves M., Paiva B.R., Leite P.E.C., Sanz C.L. (2024). Effects of propolis supplementation on gut microbiota and uremic toxin profiles of patients undergoing hemodialysis. Toxins.

[151] Hallajzadeh J., Milajerdi A., Amirani E., Attari V.E., Maghsoudi H., Mirhashemi S.M. (2021). Effects of propolis supplementation on glycemic status, lipid profiles, inflammation and oxidative stress, liver enzymes, and body weight: A systematic review and meta-analysis of randomized controlled clinical trials. J. Diabetes Metab. Disord..

[152] Kanazashi M., Iida T., Nakanishi R., Tanaka M., Ikeda H., Takamiya N., Maeshige N., Kondo H., Nishigami T., Harada T. (2023). Brazilian propolis intake decreases body fat mass and oxidative stress in community-dwelling elderly females: A randomized placebo-controlled trial. Nutrients.

[153] Moayedi F., Taghian F., Dehkordi K.J., Hosseini S.A. (2023). Cumulative effects of exercise training and consumption of propolis on managing diabetic dyslipidemia in adult women: A single-blind, randomized, controlled trial with pre-post-intervention assessments. J. Physiol. Sci..

[154] Ochoa-Morales P.D., González-Ortiz M., Martínez-Abundis E., Pérez-Rubio K.G., Patiño-Laguna A.D.J. (2023). Anti-hyperglycemic effects of propolis or metformin in type 2 diabetes mellitus A randomized controlled trial. Int. J. Vitam. Nutr. Res..

[155] Nikbaf-Shandiz M., Tutunchi H., Khoshbaten M., Bonab H.N., Ebrahimi-Mameghani M. (2022). Propolis supplementation in obese patients with non-alcoholic fatty liver disease: Effects on glucose homeostasis, lipid profile, liver function, anthropometric indices and meta-inflammation. Food Funct..

[156] Samadi N., Mozaffari-Khosravi H., Rahmanian M., Askarishahi M. (2017). Effects of bee propolis supplementation on glycemic control, lipid profile and insulin resistance indices in patients with type 2 diabetes: A randomized, double-blind clinical trial. J. Integr. Med..

[157] El-Sharkawy H.M., Anees M.M., Van Dyke T.E. (2016). Propolis improves periodontal status and glycemic control in patients with type 2 diabetes mellitus and chronic periodontitis: A randomized clinical trial. J. Periodontol..

[158] Zhao L.T., Pu L.L., Wei J.Y., Li J.H., Wu J.Q., Xin Z.H., Gao W.N., Guo C.J. (2016). Brazilian green propolis improves antioxidant function in patients with type 2 diabetes mellitus. Int. J. Environ. Res. Public Health.

[159] Liu T., Li Z.Z., Xie Q.Y., Shu X., Yu W.J., Cao J.Z., Luo L.P. (2024). Long-term propolis intake-induced liver lipid remodeling in mice: Effects on phospholipid-to-glycerolipid metabolism and free fatty acid-mediated thermogenesis. J. Agric. Food Chem..

[160] Oršolić N., Landeka Jurčević I., Đikić D., Rogić D., Odeh D., Balta V., Perak Junaković E., Terzić S., Jutrić D. (2019). Effect of propolis on diet-induced hyperlipidemia and atherogenic indices in mice. Antioxidants.

[161] Emil A.B., Hassan N.H., Ibrahim S., Hassanen E.I., Eldin Z.E., Ali S.E. (2024). Propolis extract nanoparticles alleviate diabetes-induced reproductive dysfunction in male rats: Antidiabetic, antioxidant, and steroidogenesis modulatory role. Sci. Rep..

[162] Hafez S.M., Ibrahim H.F., Abdelmohsen S.R., Yasin N.A.E., Abouelela Y.S., Aboelsoud H.A. (2024). The potential protective effect of propolis on diabetic nephropathy induced by streptozotocin in adult albino rats. Ultrastruct. Pathol..

[163] Nna V.U., Abu Bakar A.B., Md Lazin M., Mohamed M. (2018). Antioxidant, anti-inflammatory and synergistic anti-hyperglycemic effects of Malaysian propolis and metformin in streptozotocin-induced diabetic rats. Food Chem. Toxicol..

[164] Usman U.Z., Bakar A.B.A., Mohamed M. (2018). Propolis improves pregnancy outcomes and placental oxidative stress status in streptozotocin-induced diabetic rats. BMC Complement. Altern. Med..

[165] Zhu W., Chen M., Shou Q., Li Y., Hu F. (2011). Biological activities of chinese propolis and brazilian propolis on streptozotocin-induced type 1 diabetes mellitus in rats. Evid. Based Complement. Altern. Med..

[166] Hassan O.S., Megahed M.A., Ghazal N.A. (2025). White adipose tissues and skeletal muscles as a target of chrysin during the treatment of obesity in rats. Sci. Rep..

[167] Zhang Z.Y., He Z.Z., Wang X.Y., Huang B.Y., Zhang W.R., Sha Y.W., Pang W.J. (2025). A natural small molecule pinocembrin resists high-fat diet-induced obesity through GPR120-ERK1/2 pathway. J. Nutr. Biochem..

[168] Siddiqui M.A., Badruddeen, Akhtar J., Uddin S., Chandrashekharan S.M., Ahmad M., Khan M.I., Khalid M. (2022). Chrysin modulates protein kinase IKKε/TBK1, insulin sensitivity and hepatic fatty infiltration in diet-induced obese mice. Drug Develop. Res..

[169] Yuvaraj S., Vasudevan V., Puhari S.S.M., Sasikumar S., Ramprasath T., Selvi M.S., Selvam G.S. (2024). Chrysin reduces heart endoplasmic reticulum stress-induced apoptosis by inhibiting PERK and Caspase 3-7 in high-fat diet-fed rats. Mol. Biol. Rep..

[170] Nishikawa S., Hyodo T., Aoyama H., Miyata R., Kumazawa S., Tsuda T. (2020). Artepillin C, a Key component of Brazilian Propolis, induces thermogenesis in inguinal white adipose tissue of mice through a creatine-metabolism-related thermogenic pathway (vol 68, pg 1007, 2020). J. Agr. Food Chem..

[171] Zhang J.F., Wu J.W., Shi X.C., Li D.F., Yang S.Z., Zhang R.X., Xia B., Yang G.S. (2024). A propolis-derived small molecule tectochrysin ameliorates type 2 diabetes in mice by activating insulin receptor β. Mol. Nutr. Food Res..

[172] Zhao Y., Li B., Liu J., Chen L., Teng H. (2024). Galangin prevents against ethanol-induced intestinal barrier dysfunction and NLRP3 inflammasome activation via NF-κB/MAPK signaling pathways in mice and Caco-2 cells. J. Agric. Food Chem..

[173] Zhong X.C., Liu Y.M., Gao X.X., Krausz K.W., Niu B., Gonzalez F.J., Xie C. (2023). Caffeic acid phenethyl ester suppresses intestinal FXR signaling and ameliorates nonalcoholic fatty liver disease by inhibiting bacterial bile salt hydrolase activity. Acta Pharmacol. Sin..

[174] Cai W., Xu J., Li G., Liu T., Guo X., Wang H., Luo L. (2020). Ethanol extract of propolis prevents high-fat diet-induced insulin resistance and obesity in association with modulation of gut microbiota in mice. Food Res. Int..

[175] Aliakbarian M., Jazinaki M.S., Bahari H., Rashidmayvan M., Golafrouz H., Khodashahi R., Pahlavani N. (2024). Effects of propolis consumption on liver enzymes and obesity indices in adults: A systematic review and dose-response meta-analysis. Curr. Dev. Nutr..

[176] Koya-Miyata S., Arai N., Mizote A., Taniguchi Y., Ushio S., Iwaki K., Fukuda S. (2009). Propolis prevents diet-induced hyperlipidemia and mitigates weight gain in diet-induced obesity in mice. Biol. Pharm. Bull..

[177] Sakai T., Ohhata M., Fujii M., Oda S., Kusaka Y., Matsumoto M., Nakamoto A., Taki T., Nakamoto M., Shuto E. (2017). Brazilian green propolis promotes weight loss and reduces fat accumulation in C57BL/6 mice fed a high-fat diet. Biol. Pharm. Bull..

[178] Washio K., Shimamoto Y., Kitamura H. (2015). Brazilian propolis extract increases leptin expression in mouse adipocytes. Biomed. Res..

[179] Christoper A., Gunawan E., Herman H., Abdulah R., Zulhendri F., Popova M., Trusheva B., Bankova V., Lesmana R. (2025). The role of Indonesian stingless bee *Geniotrigona thoracica* propolis extract in ameliorating obesity and modulating inflammation and autophagy process in the brain after palmitic-based high-fat diet exposure. J. Funct. Foods.

[180] Lisbona González M.J., Reyes Botella C., Muñoz Soto E., Olmedo Gaya M.V., Moreno Fernández J., Díaz Castro J. (2021). Body composition, mineral metabolism, and endocrine function of adipose tissue: Influence of a nutritional supplement of propolis. Nutr. Hosp..

[181] Rahayu N., Nur A.N.U., Diniyah A.B., Fendi F. (2020). Propolis and honey trigona decrease leptin levels of central obesity patients. Enferm. Clin..

[182] Sun H., Saeedi P., Karuranga S., Pinkepank M., Ogurtsova K., Duncan B.B., Stein C., Basit A., Chan J.C.N., Mbanya J.C. (2022). IDF Diabetes Atlas: Global, regional and country-level diabetes prevalence estimates for 2021 and projections for 2045. Diabetes Res. Clin. Pract..

[183] Afsharpour F., Javadi M., Hashemipour S., Koushan Y., Haghighian H.K. (2019). Propolis supplementation improves glycemic and antioxidant status in patients with type 2 diabetes: A randomized, double-blind, placebo-controlled study. Complement. Ther. Med..

[184] Chen L.H., Chien Y.W., Chang M.L., Hou C.C., Chan C.H., Tang H.W., Huang H.Y. (2018). Taiwanese green propolis ethanol extract delays the progression of type 2 diabetes mellitus in rats treated with streptozotocin/high-fat diet. Nutrients.

[185] Cunha G.A.D., Carlstrom P.F., Franchin M., Alencar S.M., Ikegaki M., Rosalen P.L. (2023). A systematic review of the potential effects of propolis extracts on experimentally-induced diabetes. Planta Med..

[186] Gao W., Pu L., Wei J., Yao Z., Wang Y., Shi T., Zhao L., Jiao C., Guo C. (2018). Serum antioxidant parameters are significantly increased in patients with type 2 diabetes mellitus after consumption of chinese propolis: A randomized controlled trial based on fasting serum glucose level. Diabetes Ther..

[187] Laaroussi H., Bakour M., Ousaaid D., Aboulghazi A., Ferreira-Santos P., Genisheva Z., Teixeira J.A., Lyoussi B. (2020). Effect of antioxidant-rich propolis and bee pollen extracts against D-glucose induced type 2 diabetes in rats. Food Res. Int..

[188] Sameni H.R., Ramhormozi P., Bandegi A.R., Taherian A.A., Mirmohammadkhani M., Safari M. (2016). Effects of ethanol extract of propolis on histopathological changes and anti-oxidant defense of kidney in a rat model for type 1 diabetes mellitus. J. Diabetes Investig..

[189] Wong C.N., Lee S.K., Liew K.B., Chew Y.L., Chua A.L. (2025). Mechanistic insights into propolis in targeting type 2 diabetes mellitus: A systematic review. Planta Med..

[190] Farida S., Pratiwi D.K., Sari M., Andriani A.M., Dewi R., Winarti W., Ananda R., Azzahra T.A., Rahmawati S.I., Putra M.Y. (2023). *In vitro* study on antidiabetic and antihypertensive activities of ethanolic extract of propolis of Indonesian stingless bee Tetragonula sapiens. J. King Saud. Univ. Sci..

[191] Aga M., Arai N., Ohashi E., Ariyasu T., Arai S., Iwaki K., Ohta T., Fukuda S. (2009). Propolis enhances adipocyte differentiation and prevents insulin resistance in 3T3-L1 cells (Propolis improves insulin resistance *in vitro*). Nippon Shokuhin Kagaku Kogaku Kaishi.

[192] Sarikurkcu H.H.S.O., Demir I., Korkmaz E., Yildiz M., Ozen T. (2024). Investigating the hepatoprotective and antidiabetic properties of cryogenically pulverized Turkish propolis water extracts in streptozotocin-induced diabetic rats. S. Afr. J. Bot..

[193] El Menyiy N., Al-Wali N., El Ghouizi A., El-Guendouz S., Salom K., Lyoussi B. (2019). Potential therapeutic effect of Moroccan propolis in hyperglycemia, dyslipidemia, and hepatorenal dysfunction in diabetic rats. Iran. J. Basic Med. Sci..

[194] Kitamura H., Naoe Y., Kimura S., Miyamoto T., Okamoto S., Toda C., Shimamoto Y., Iwanaga T., Miyoshi I. (2013). Beneficial effects of Brazilian propolis on type 2 diabetes in ob/ob mice: Possible involvement of immune cells in mesenteric adipose tissue. Adipocyte.

[195] Karimian J., Hadi A., Pourmasoumi M., Najafgholizadeh A., Ghavami A. (2019). The efficacy of propolis on markers of glycemic control in adults with type 2 diabetes mellitus: A systematic review and meta-analysis. Phytother. Res..

[196] Mosallanezhad Z., Clark C., Bahreini F., Motamed Z., Mosallanezhad A., Hosseini S.F., Shaban-Khalaf A., Sohrabi Z. (2021). Effect of propolis on glycemic control in patients with type 2 diabetes: An updated systematic review and meta-analysis of randomized controlled trials. Nutr. Food Sci..

[197] Yousefi M., Hashemipour S., Shiri-Shahsavar M.R., Koushan Y., Hosseini H.K. (2023). Reducing the inflammatory interleukins with anti-inflammatory and antioxidant effects of propolis in patients with type 2 diabetes: Double-blind, randomized controlled clinical trial. Via Medica.

[198] Sani L., Cardinault N., Astier J., Darmon P., Landrier J.F. (2023). Poplar propolis improves insulin homeostasis in non-diabetic insulin-resistant volunteers with obesity: A crossover randomized controlled trial. Antioxidants.

[199] Sartori D.R.S. (2009). Propolis Effect on Streptozotocin-Induced Diabetic Rats. Ph.D. Thesis.

[200] Salas A.L., Mehltreter M.I., Eugenia Orqueda M., Correa Uriburu F.M., García M.E., Pérez M.J., Alvarez M.D.L.A., Ponessa G.I., Maldonado L.M., Zampini I.C. (2020). Zuccagnia-type propolis from Argentina: A potential functional ingredient in food to pathologies associated to metabolic syndrome and oxidative stress. J. Food Sci..

[201] Wang L., Zhou L., Liu S., Liu Y., Zhao J., Chen Y., Liu Y. (2023). Artepillin C time-dependently alleviates metabolic syndrome in obese mice by regulating CREB/CRTC2-BMAL1 signaling. Nutrients.

[202] Ahmed Y.B., Jasim S., Mustafa Y.F., Hussien B., Diwan T.M., Singh M. (2024). The effects of propolis supplementation on lipid profiles in adults with metabolic syndrome and related disorders: A systematic review and meta-analysis of randomized controlled trials. Hum. Nutr. Metab..

[203] Gholami Z., Maracy M.R., Paknahad Z. (2024). The effects of MIND diet and propolis supplementation on metabolic syndrome: A randomized controlled clinical trial. Heliyon.

[204] Sajjadi S.S., Bagherniya M., Soleimani D., Siavash Dastjerdi M., Askari G. (2023). Effect of propolis on mood, quality of life, and metabolic profiles in subjects with metabolic syndrome: A randomized clinical trial. Sci. Rep..

[205] Younossi Z.M., Koenig A.B., Abdelatif D., Fazel Y., Henry L., Wymer M. (2016). Global epidemiology of nonalcoholic fatty liver disease-Meta-analytic assessment of prevalence, incidence, and outcomes. Hepatology.

[206] Abd-Elrazek A.M., Ibrahim S.R., El-dash H.A. (2022). The ameliorative effect of Apium graveolens & curcumin against Non-alcoholic fatty liver disease induced by high fructose-high fat diet in rats. Future J. Pharm. Sci..

[207] Jin X., Wang K., Li Q., Tian W., Xue X., Wu L., Hu F. (2017). Antioxidant and anti-inflammatory effects of Chinese propolis during palmitic acid-induced lipotoxicity in cultured hepatocytes. J. Funct. Foods.

[208] Liu P.P., Wu P.X., Yang B.D., Wang T.Q., Li J.D., Song X.H., Sun W.L. (2021). Kaempferol prevents the progression from simple steatosis to non-alcoholic steatohepatitis by inhibiting the NF-KB pathway in oleic acid-induced HepG2 cells and high-fat diet-induced rats. J. Funct. Foods.

[209] Nazari-Bonab H., Nikbaf-Shandiz M., Tutunchi H., Ebrahimi-Mameghani M. (2024). Effects of propolis supplementation on prooxidant-antioxidant balance, oxidative stress biomarkers, and body composition in obese patients with NAFLD: A double-blind randomized controlled clinical trial. Health Promot. Perspect..

[210] Pai S.A., Munshi R.P., Panchal F.H., Gaur I.S., Juvekar A.R. (2019). Chrysin ameliorates nonalcoholic fatty liver disease in rats. Naunyn-Schmiedeberg’s Arch. Pharmacol..

[211] Kismet K., Ozcan C., Kuru S., Gençay Çelemli Ö.G., Celepli P., Şeneş M., Guclu T., Sorkun K., Hücümenǒglu S., Besler T. (2017). Does propolis have any effect on non-alcoholic fatty liver disease?. Biomed. Pharmacother..

[212] Ogawa T., Terada T. (2024). The beneficial effect of brazilian propolis for liver damage through endoplasmic reticulum stress. Biol. Pharm. Bull..

[213] Tutunchi H., Arefhosseini S., Ebrahimi-Mameghani M. (2023). Clinical effectiveness of a-lipoic acid, myo-inositol and propolis supplementation on metabolic profiles and liver function in obese patients with NAFLD: A randomized controlled clinical trial. Clin. Nutr. ESPEN.

