# Supplementary material for: Propolis in Obesity and Related Metabolic Disorders: Mechanistic and Clinical Insights—A Scoping Review
Source: Nutrients. 2026 Mar 3;18(5):826. doi: 10.3390/nu18050826 (PMC12987181; doi:10.3390/nu18050826)
Supplement: Supplementary file 1 [file nutrients-18-00826-s001.zip › Supplementary_Table_S1_Search_Strategy.pdf]

Supplementary Materials

Supplementary Table S1. Detailed Electronic Search Strategy

**Database**	**PubMed/MEDLINE (National Library of Medicine)**
Date of Last Search	12 July 2025
Time Frame	From database inception to 12 July 2025
Language Restriction	No restrictions applied
Document Type Restriction	No restrictions applied
Notes	Search terms were adapted for Scopus and Web of Science.

Full Search Strategy (PubMed)

```
(
  "Propolis"[Mesh] OR propolis[tiab] OR "bee glue"[tiab]
)
AND
(
  "Obesity"[Mesh] OR obesity[tiab] OR obese[tiab] OR adiposity[tiab]
  OR overweight[tiab]
  OR "Body Weight"[Mesh]
  OR "Body Mass Index"[Mesh]
  OR "Metabolic Diseases"[Mesh]
  OR "Metabolic Syndrome"[Mesh] OR "metabolic syndrome"[tiab]
  OR "Insulin Resistance"[Mesh] OR "insulin resistance"[tiab]
  OR "Diabetes Mellitus, Type 2"[Mesh] OR "type 2 diabetes"[tiab] OR T2DM[tiab]
  OR "Non-alcoholic Fatty Liver Disease"[Mesh] OR NAFLD[tiab] OR MASLD[tiab]
  OR dyslipidemia[tiab] OR hyperlipidemia[tiab]
  OR "Lipid Metabolism"[Mesh] OR "lipid metabolism"[tiab]
  OR adipogenesis[tiab]
  OR inflammation[tiab] OR "oxidative stress"[tiab]
  OR "gut microbiota"[tiab] OR microbiome[tiab]
)
```
